# The porphyran degradation system is complete, phylogenetically and geographically diverse across the gut microbiota of East Asian populations

**DOI:** 10.1371/journal.pone.0329457

**Published:** 2025-08-01

**Authors:** Laure Ségurel, Thirumalai Selvi Ulaganathan, Sophie Mathieu, Mélanie Loiodice, Laurent Poulet, Sophie Drouillard, Miroslaw Cygler, William Helbert

**Affiliations:** 1 Laboratoire de Biométrie et Biologie Evolutive, UMR 5558, CNRS - UCB Lyon 1, Villeurbanne cedex, France; 2 Department of Biochemistry, University of Saskatchewan, Saskatoon, Saskatchewan, Canada; 3 CERMAV, CNRS and Grenoble Alpes Université, BP53, 38000 Grenoble Cedex 9, France; University of Nebraska-Lincoln, UNITED STATES OF AMERICA

## Abstract

The human gut microbiota can acquire new catabolic functions by integrating genetic material coming from the environment, for example from food-associated bacteria. An illustrative example of that is the acquisition by the human gut microbiota of Asian populations of genes coming from marine bacteria living on the surface of red algae that are incorporated into their diet when eating maki-sushi. To better understand the function and evolution of this set of algal genes corresponding to a polysaccharide utilization locus (PUL) dedicated to the degradation of porphyran, the main polysaccharide of the red algae *Porphyra sp*., we characterized it biochemically, assessed its genetic diversity and investigated its geographical distribution in large public worldwide datasets. We first demonstrated that both methylated and unmethylated fractions are catabolized without the help of external enzymes. By scanning the genomic data of more than 10,000 cultivated isolates as well as metagenomic data from more than 14,000 worldwide individuals, we found that the porphyran PUL is present in 17 different *Phocaeicola*/*Bacteroides* species (including 12 species that were not known to carry it), as well as in two *Parabacteroides* species and two genera from the Bacillota phylum, highlighting multiple lateral transfers within the gut microbiota. We then analyzed the prevalence of this porphyran PUL across 32 countries and showed that it exists in appreciable frequencies (>1%) only in East Asia (Japan, China, Korea). Finally, we identified three major PUL haplotypes which frequencies significantly differ between these East Asian countries. This geographic structure likely reflects the rate of bacterial horizontal transmission between individuals.

## Introduction

The impact of diet on the composition of the bacterial communities inhabiting the human gut is now well documented [1,2]. For example, the ratio of Firmicutes (now Bacillota) to Bacteroidetes (now Bacteroidota) decreases with calorie-restricted or high-fiber diets, and the abundance of *Prevotella* is associated with the intake of carbohydrates while that of *Bacteroides* is associated with animal protein and fat [3–5]. In parallel, the gut microbiota can also acquire new functional capacities by horizontal transfer of genetic material between species of the gut microbiota but also from species found in the food ingested by the host [6–8]. One illustrative example of how the food participates in shaping the human gut microbiota, at the molecular level, is the discovery of the horizontal gene transfer of a set of enzymes involved in the degradation of porphyran, the cell wall polysaccharide of the red algae *Porphyra sp.* used to prepare the maki-sushi, from a marine species living at the surface of the algae to the gut bacteria *Bacteroides plebeius* (now *Phocaeicola plebeius*) found in the Japanese gut microbiota [9]. Since this discovery, other algal polysaccharides degradation systems (i.e., agarose, alginate, carrageenan), likely acquired by lateral transfer from marine organisms, have also been studied [9–13].

Porphyran is an agar-type polysaccharide made of two disaccharides repetition: agarobiose and porphyranobiose decorated up to 64% by methyl groups [14,15] (S1A Fig). In *B. plebieus*, the genes encoding the enzymes degrading porphyran are co-localized and co-regulated in a so-called “polysaccharide utilizing loci” (PUL). The porphyran PUL identified in *B. plebieus* contains genes encoding two β-porphyranases (BpGH16B, Bacple_01689; BpGH86A, Bacple_01693) and two β-agarases (BpGH16A; Bacple_01670; BpGH86B, Bacple_01694) [9,16] (S1B Fig). The oligosaccharides resulting from the degradation of porphyran by these enzymes are then degraded further by exo-acting enzymes including sulfatase (BpS1_11, Bacple_01701), α-L-galactosidase (BpGH29, Bacple_01702) and β-D-galactosidase (BpGH2C, Bacple_01706) produced by *B. plebeius* or another agarolytic strain such as *Bacteroides uniformis* [13] (S1B Fig). Altogether, the many enzymes of the porphyran PUL (i.e., glycoside hydrolases and sulfatases) have been characterized, explaining the degradation process of the agarose components and the non-methylated fractions of porphyran by *B. plebieus* [9,16,17] (S1B Fig). However, the enzymes involved in the degradation of abundant fraction of the methylated porphyran have not been discovered yet.

Even though the fronds of *Porphyra sp*. are nowadays mostly consumed by Asian populations for their taste and nutritional value [18], the most ancient proof that humans harvested and stored *Porphyra sp.* was found in the archaeological site of Monte Verde (Chile) occupied by humans 12,300 years ago [19]. The traditional use of *Porphyra spp.* by the first nations living along the Pacific coast of North America was also documented by ethnobotanists [20–23]. In South America, pre-Inca and Inca populations also incorporated red algae in their diet [24]. Furthermore, Europeans populations (from Spain to Scotland to Lithuania) have recently been found to have consumed seaweed from the Neolithic transition thousands of years ago up to the early Middle Ages [25], raising the question of whether these horizontally-transferred genes are found in different places across the globe. Originally, the porphyran PUL was identified in individuals from Japan. Since then, Pudlo et al [13], even though they did not report quantitative values for the porphyran PUL prevalence, showed that it was found both in East Asia (China, Japan) but also in Western countries. A detailed observation of their reported data (their Fig 7) shows that it is around a frequency of 30% in both Japan and China, while being at around 2% in Western countries and absent in Tanzania or Brazil (survey of a dataset of 2,440 individuals). Furthermore, among Western countries, the porphyran PUL prevalence seems to be in low frequency in individuals living in the USA (HMP project), and completely absent in individuals living in Denmark or Spain (Metahit project). As we expect a small proportion of individuals currently living in the USA to be of East Asian origin, it is not clear whether that low prevalence reflects only recent immigration, or whether the porphyran PUL has also been around for some time in USA. However, more data (covering more than 14,000 individuals) is now available in the literature to tackle that question in more detail.

**Fig 1:**
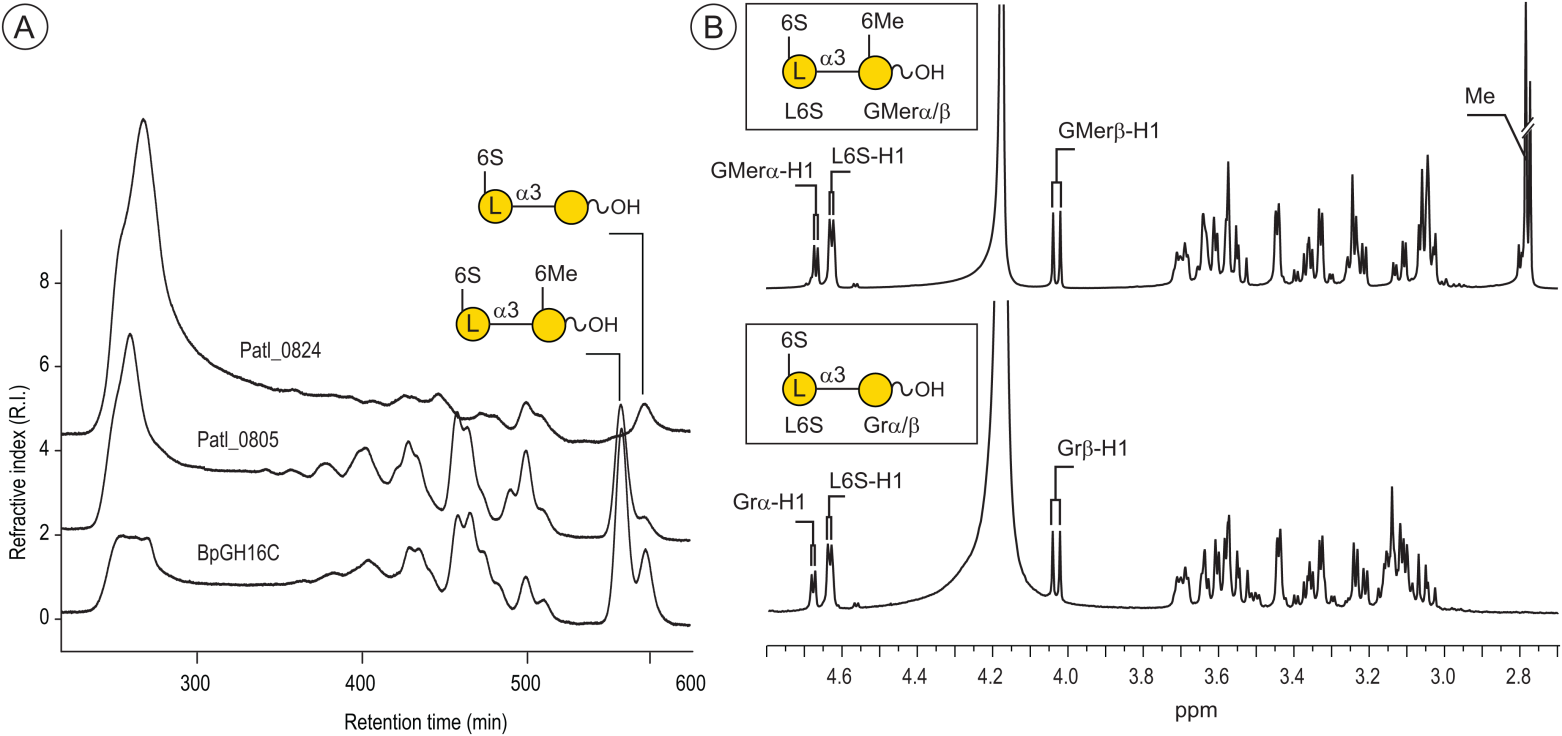
Degradation of porphyran by BpGH16C (Bacple_01703). **A**) Size exclusion chromatography of the degradation products of porphyran incubated with the methyl-6O-β-porphyranase BpGH16C. The degradation profile was compared with the two *P. atlantica* T6c porphyranases grouped in the GH16_12 (Patl_0824) and GH16_14 (Patl_0805) sub-families. **B**) ^1^H NMR of the disaccharides end-products of BpGH16C.

**Fig 2:**
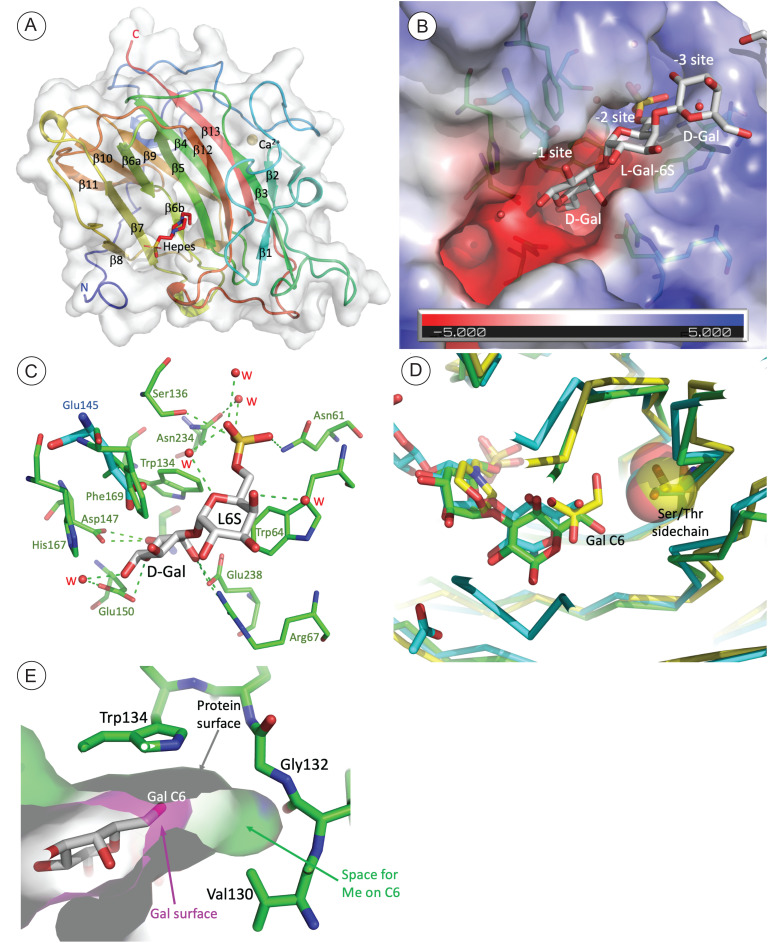
Crystal structure of BpGH16C (Bacple_01703). **A**) Chain A of asymmetric unit is shown in cartoon representation and colored in spectrum from N- to C-terminals. The HEPES molecule in the active site is shown as stick representation. The N- and C-terminals are marked, and the strands are labeled in their order in the sequence. **B**) The substrate binding site in the mutant BpGH16C-E145L complexed with the D-Gal-β4-6-sulfate-L-Gal disaccharide end-product. The semitransparent surface of the protein is colored by the electrostatic potential. The residues forming the site are shown in stick representation. The sulfate group from L-galactose 6 sulfate is docked into a positively charged pocket. **C**) The hydrogen bonds between the disaccharide in substrate binding site residues. Several H-bonds are bridged by water molecules (W). Glu145 (in blue and thicker bonds) from the native structure is superimposed on the Leu145 in the mutants. **D**) Structure comparison of BpGH16C (green), PorA (PDB id-3ILF) (magenta) and PorB (PDB id- 3JUU) (yellow) showing the active site region. The single mutation of Ser129/Thr137 (PorA/PorB) (sidechains shown as spheres) to Gly132 in BpGH16C provides space for accommodating a methyl group at C6 on L-galactose residue. **E**) A cross-section of the surface representation of the Gal sugar and the cavity in the binding site near Gly132. There is free space within the cavity sufficient to accommodate the methyl group of a methylated porphyran.

**Fig 3:**
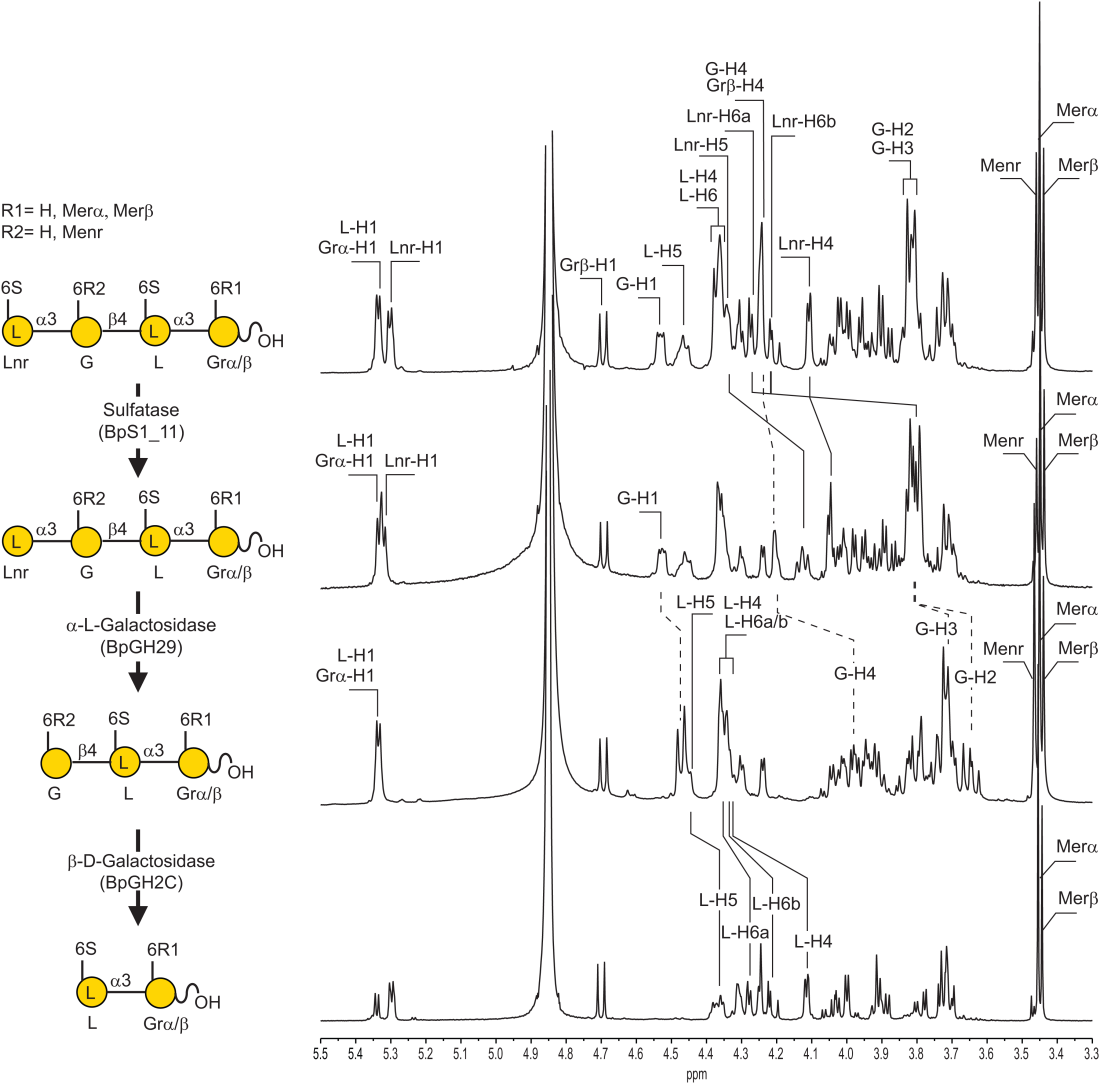
Catabolism of oligo-porphyran. ^1^H NMR recorded after sequential incubation of the methylated tetrasaccharide end-products of the 6-O-methyl-β-porphyranase BpGH16C with the sulfatase BpS1_11 followed by the β-L-galactosidase BpGH29 and the β-D-galactosidase BpGH2C.

**Fig 4:**
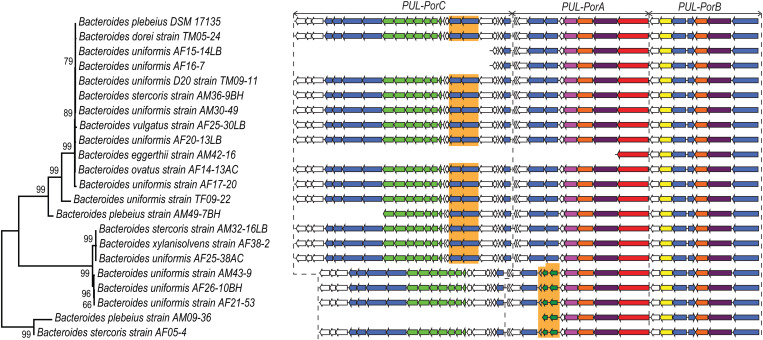
Diversity of the porphyran PUL organization. Gene organization of the porphyran degradation system of *B. plebieus* compared with other identified human gut bacteria. Phylogenetic tree was calculated using concaneted gene sequences of *PUL-PorB*. PUL organizations of each strain was indicated based on the available sequencing data.

**Fig 5:**
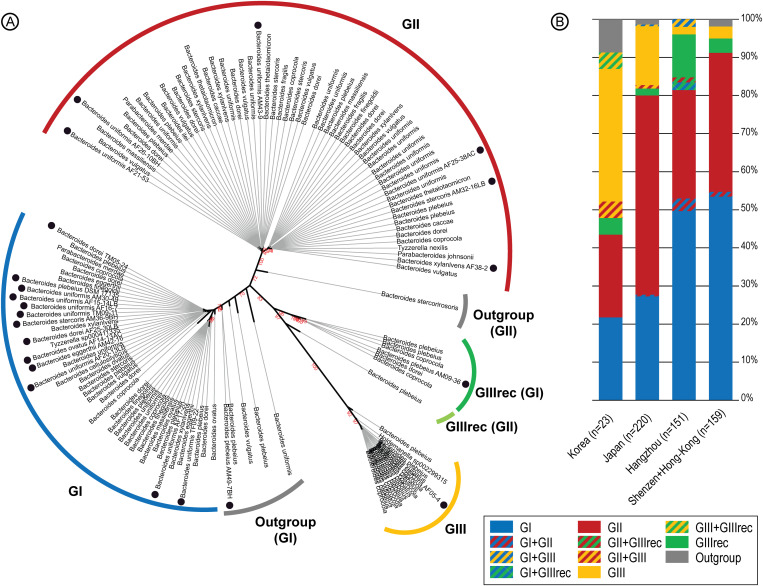
Distribution of the *PUL PorB* groups in assembled metagenome. **A**) Phylogenetic tree calculated with the concatenated protein sequences of *PUL-PorB* recorded in non-redundant human gut bacteria including isolated strains (●). The observed clades were used to create groups of homologous porphyran PUL *PUL-PorB* (GI, GII, GIII and GIIIrec) and divergent *PUL-PorB* (Outgroup). **B**) Distribution of the different group of the *PUL-PorB* in human gut metagenome assembly of South east and North East Chinese, Korea and Japanese populations.

**Fig 6:**
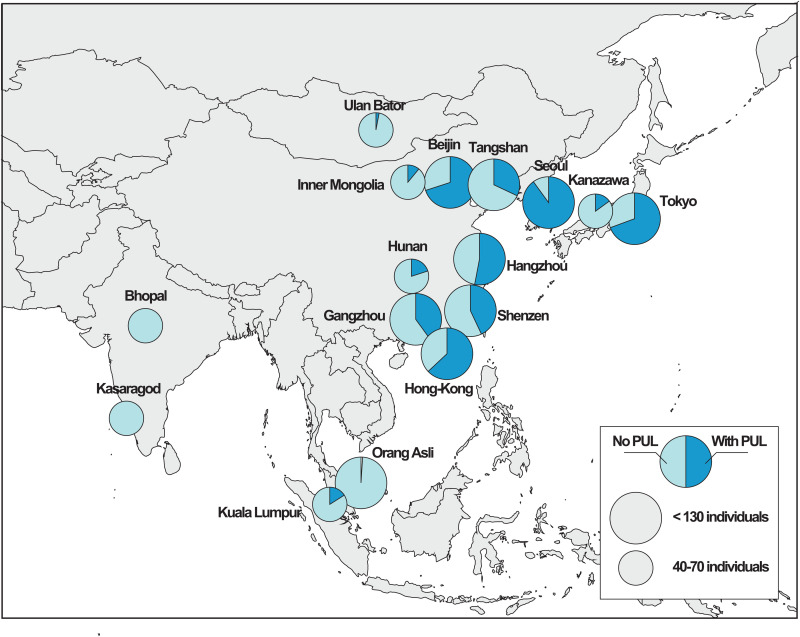
Proportion of individuals with short reads mapping to the 54-nucleotides probes specific to the *PUL-PorB* in Asia (PUL-positive individuals), grouped by city. Some geographic coordinates have been adjusted so that each point is visible.

**Fig 7:**
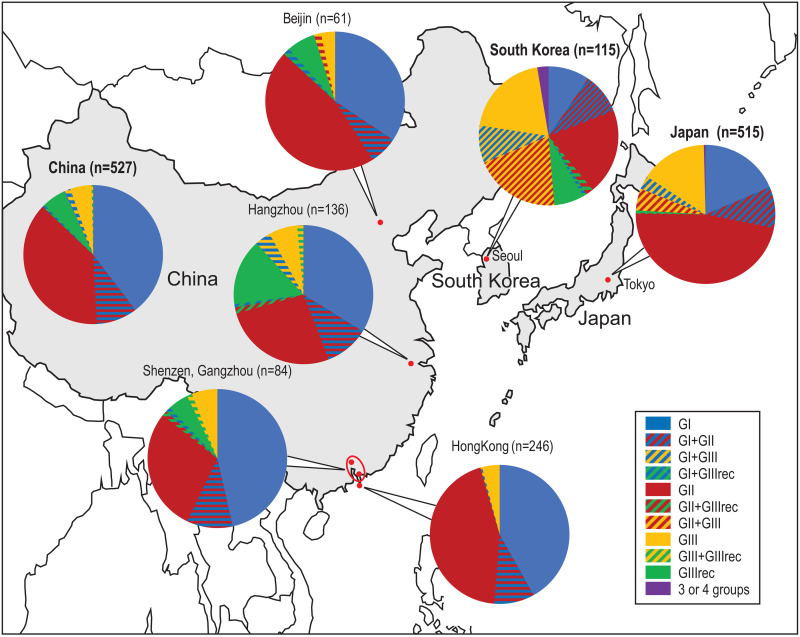
Distribution of the various porphyran PUL among Chinese, Japanese and Korean populations. The pie charts derive from the histogram presented in S9 Fig obtained from the analyses of short read sequencing of Chinese, Japanese and Korean metagenomics data.

The number of human gut species carrying this metabolic capacity, allowing to make hypotheses about the origin of the algal degradation system in the human gut, is not fully resolved either. A previous study of *in vitro* cultivation of stool samples of 240 donors in the presence of porphyran indeed showed that four other *Phocaeicola*/Bacteroides species (*B. fragilis, B. ovatus, B. thetaiotaomicron* and *B. xylanisolvens*), as well as *Faecalicatena contorta* (from the Bacillota phylum), were able to grow on porphyran [13], but a large-scale survey of existing gut bacterial genomes has not been performed yet.

We thus aimed to continue the detailed analysis of the porphyran PUL of *B. plebeius* by biochemically studying the uncharacterized glycoside hydrolases BpGH16C (Bacple_01703) and BpGH2C (Bacple_01706) and by completing the structural data of the sulfatase BpS1_11. Our investigation revealed that the complete degradation of methylated and non-methylated fractions of porphyran can be obtained by the porphyran PUL of *B. plebieus* without the help of another strain as previously suggested [13]. Therefore, the evolution and distribution of functional porphyran PUL in human population is constraint by the occurrence of all the necessary enzymes. In this context, we further took advantage of the increasingly large genomic datasets available in the literature to survey the genomes of more than 10,000 gut isolates, as well as the metagenomes of 14,000 worldwide individuals, in order i) to evaluate the number of gut bacterial species carrying the porphyran PUL, ii) to identify the number of countries where the porphyran PUL is present, and iii) to characterize the genetic diversity of porphyran PUL and its geographical differentiation.

## Results

### Biochemical and molecular characterization of the endo-acting 6O-methyl-porphyranase BpGH16C (Bacple_01703)

The gene Bacple_01703 encodes a glycoside hydrolase of the GH16 family (BpGH16C), which includes characterized agarases in sub-families GH16_15-16, and β-porphyranases in sub-families GH16_11-12 [26]. BpGH16C was classified in the GH16_14 sub-family that contains one characterized β-agarase (*Vibrio sp*. strain PO-303, BAF62129.1) [27], one endo-6O-methyl-β-porphyranase (*Wenyingzhuangia fucanilytica*, WP_068825734.1) [28] and one β-porphyranase observed in the red algal *Chondrus crispus* [29]. We assayed BpGH16C on various marine polysaccharides including carrageenan, agarose and porphyran. Only porphyran was degraded, leading to the production of a series of oligosaccharides characteristic of endo-acting glycoside hydrolase (Fig 1A). The structural characterization by NMR of the end-products purified by chromatography [30] revealed the occurrence of a methyl group on the β-linked D-galactose residue (Fig 1B), demonstrating that BpGH16C is an endo-6O-methyl-β-porphyranase able to accommodate the methyl group of porphyran in its active site. For comparison, we have examined the substrate specificities of one another predicted GH16_14 endo-6O-methyl-β-porphyranase from *Paraglaciecola atlantica* T6c (Patl_0824, ABG39352.1) presenting 51.1% identity with the BpGH16C and one GH16_12 β-porphyranase (Patl_0805, ABG39333.1) homologous to BpGH16B. Analyses of the degradation products confirmed the different porphyranase specificities (Fig 1A).

We determined the crystal structure of BpGH16C at 1.9 Å resolution (PDB ID 8EP4) (Fig 2A). The enzyme has a β-sandwich jelly-roll fold with two stacking antiparallel β-sheets typical of proteins from GH16 family [26] and like in most GH16, a Ca^2+^ is bound on the convex side of the β-sandwich [31]. One molecule of Hepes from the crystallization buffer bound in the center of the cleft, with the 2-hydroxyethyl end buried into the molecule interior and the sulfate group exposed on protein surface (Fig 2A). To get a better understanding of substrate binding and catalysis, we crystallized the E145L inactive mutant with the tetrasaccharide L-α-6O-sulfate-Gal-(1 → 3)-D-β-Gal-(1 → 4)-L-α-6O-sulfate-Gal-(1 → 3)-D-a/β-Gal and determined its structure at 1.8 Å resolution (PDB ID 8EW1). An electron density was present within the groove in all the three independent molecules in the asymmetric unit fitted a D-β-Gal-(1 → 4)-L-α-6O-sulfate-Gal-(1 → 3)-D-β-Gal trisaccharide (Fig 2B). The electron density for the D-Gal on the nonreducing end is weaker than for the first two residues and no density is observed for the fourth residue indicating its mobility in the crystal. The residues correspond to the −1, −2 and −3 positions according to the standard nomenclature [32].

The trisaccharide sits edge-on in the groove with C5 substituent of the ring in the −1 site directed toward the bottom of the cleft while O1 and O2 hydroxyls point toward the solvent. Trp134 stacks against hydrophobic side of the −1 residue while Arg67 and Glu238 hydrogen bond to the O4 hydroxyl. Residue in the −2 subsite is stacked between Trp64 and the edges of Trp143 and Phe169. Finally, the sulfate group of the −2 residue is hydrogen bonded to Asn61, Ser136 and Asn234 through a bridging water molecule. The D-Gal on the non-reducing end (−3 site) has somewhat different orientation in molecules A/B and C and makes only one bridging hydrogen bond through a bridging water (Fig 2C). The structure of the complex with a trisaccharide adds also to our understanding of the catalytic mechanism. Glu150 is indeed the closest to the O1 hydroxyl that formed the bond to the next sugar eliminated by hydrolysis, while Glu145 approaches from the opposite side of the ring relative the O1 hydroxyl (Fig 3C) and is likely helping in proper orientation of the substrate. Asp147 is directed to the bridging O1 and might be helping in catalysis.

Structural alignment of BpGH16C with three other GH16 porphyranases: porphyranase A (PDB ID 3ILF) and porphyranase B from *Zobellia galactanivorans* (PDB ID 3JUU) [9] and another porphyranase from *B. plebeius* (PDB ID 4AWD) [16] shows that the Gly132 is replaced by either a Ser or a Thr, making this pocket too small to accommodate an additional methyl group on the C6-O (Fig 2D). Sequence alignment of β-porphyranases with BpGH16C and the two other characterized 6O-methyl-β-porphyranases: Patl_0824 of (*P. atlantica* T6c, this study) and AXE80_06940 (*W. fucanilytica*) [28] confirmed that the replacement of the Ser/Thr by Gly was correlated to the accommodation of the methyl group in the −1 sub-site of the catalytic site (S2 Fig). The BpGH16C is the first crystallized 6-OMe-porphyranase that tolerates the 6-methyl group on the galactose ring. Indeed, there is an extra space in the active site at the end of the pocket that accommodates C6-OH, with its bottom formed by Gly132 (Fig 2E).

### Catabolism of oligo-methyl-porphyrans

Following the same strategy reported by Robb and co-workers [17], we have incubated the methylated oligosaccharides with the BpS1_11 sulfatase, the BpGH29 α-L-galactosidasee and the predicted BpGH2Cβ-D-galactosidase. Purified methylated and non-methylated oligo-porphyrans were incubated with the BpS1_11 sulfatase and analyses of the products by ^1^H NMR showed a strong chemical shift (about 0.1 ppm) of the signal attributed to the proton at position 6 of the galactose located at the non-reducing end, demonstrating the removal of the sulfate ester group independently of the degree of methylation of the oligosaccharides (S3 Fig).

Crystal structure of the BuS1_11 sulfatase from *B. uniformis* complexed with a di-saccharides [18] and the *B. plebeius* sulfatase BpS1_11 on its own (this study, PDB ID 7SNJ, 1.64 Å resolution) and complexed with a tetrasaccharide (this study, PDB ID 7SNO, 2.1 Å resolution) revealed that there is room in the active site to accommodate oligo-methyl-porphyrans (S4 Fig). The porphyran sulfatase BpS1_11 belongs to the formyl-glycine dependent sulfatases, requiring maturation of a cysteine or a serine into the formyl-glycine residue as an active catalytic residue. A large N-terminal domain of BS1_11 contains active site residues and forms main part of the substrate binding site. A smaller C-terminal domain completes the substrate binding site.

To map the details of substrate binding, we determined the structure of H214N inactive mutant with L-α-6O-sulfate-Gal-(1 → 3)-D-β-Gal-(1 → 4)-L-α-6O-sulfate-Gal-(1 → 3)-D-a/β-Gal tetrasaccharide substrate (PDB ID 7SNO). The electron density map clearly showed the entire tetrasaccharide bound to BpS1_11 (H214N). Following the recently proposed nomenclature [33], the tetrasaccharide binds to the 0, + 1, + 2 and +3 positions (S4 Fig). The 6S-L-Gal in position 0, which the sulfate is removed, has all three hydroxyl groups hydrogen bonded to the protein side chains: O2 to Arg271, O3 to Asp215 and Arg271, O3 to Asp345 and Arg347. The 6-sulfate group is facing Ser83 (formyl-glycine in mature enzyme) and one of its oxygens is a ligand to the Ca^2+^ ion. D-Gal in +1 position hydrogen bonds C6OH to NE2 of His133 and its O2 is bonded to the O2 of L-Gal (position 0) through a bridging water molecule. The + 2 6S-L-Gal makes only one contact, between the 6-sulfate oxygen and His428. The + 3 D-Gal extends far from the binding site and makes no contacts with the protein. However, in the crystal, this sugar hydrogen bonds from O1 and O4 as well as O5 within the ring to the guanidino group of Arg68 from a symmetry-related molecule. These interactions stabilize the + 2 and +3 sugars in the crystal. Importantly, the 6-O group of +1 D-Gal is not tightly constraint by the enzyme and there is sufficient space for the additional methyl group present in the 6-O-Me-D-Gal (S4 Fig).

We confirmed the exo-based cycle of porphyran depolymerization [17] using tetra-saccharides as starting substrate (Fig 3, S1 Table). After desulfation of the tetra-saccharides, BpGH29 exo-α-L-galactosidase was active on the methylated and non-methylated substrate demonstrating that the methyl group did not hinder the activity of the enzyme. Independently of Robb and co-workers [17], we have determined the structure of BpGH29 (PDB ID 7SNK). The superposition with their structure (PDB ID 7LJJ) shows root-mean-squares deviation of 0.6 Å, indicating that the structures that were crystallized under different conditions are virtually identical, and confirms that ordering of the loop 408–426 and small rearrangement of other surrounding loops result from the substrate binding to the active site.

Finally, we observed that the methylated or non-methylated D-galactose residue located at the non-reducing end of the trisaccharide was cleaved by the BpGH2C β-D-galactosidase (Fig 3). Altogether, the enzymology and crystallography experiments conducted in this work revealed the pathway leading to the degradation of the methylated fraction of porphyran. Our observations combined with previous investigations demonstrate that all the methylated and non-methylated fractions of porphyran are digested by the enzymes encoded by porphyran PUL of *B. plebeius*. Therefore, this PUL can catabolize autonomously the chemically complex porphyran, without the help of enzyme(s) external to this PUL.

### Phylogenetic distribution of the porphyran utilizing loci and gene organization

*B. plebeius* DSM 17135 was the first gut bacteria shown to carry the porphyran PUL. Recently, some strains of *B. fragilis*, *B. ovatus*, *B. thetaiotaomicron* and *B. xylanisolvens* were further shown to be able to grow on porphyran and to carry a > 96% identical sequence to the *B. plebeius* porphyran PUL [13]. In order to investigate in more depth whether other human gut bacteria also carry the porphyran PUL, we used BLASTn to identify homologs of the entire coding sequences of *PUL-PorA*, *PUL-PorB* and *PUL-PorC* of *B. plebeius* DSM 17135 among the 10,969 genomes of bacteria isolated from the human gut and catalogued by Almeida and co-workers [34]. We found that the PULs were present in the genomes of 22 *Phocaeicola*/*Bacteroides* strains (corresponding to at least 8 different identified species, S2 Table), including five new species not described so far in Pudlo *et al* [13]: *B. dorei*, *B. eggerthi*, *B. stercoris*, *B. uniformis* and *B. vulgatus*. All these strains came from isolates from the gut microbiome of East Asian individuals, and the same species/strains isolated from other human populations worldwide had no homologs of porphyran PULs.

While the *PUL-PorB* organization as observed in *B. plebieus* DSM 17135 was conserved in all the other strains we have identified, the gene organization of *PUL-PorA* was only conserved in 17/22 strains (Fig 4). In five other strains (*B. plebeius* strain AM09-36, *B. stercoris* strain AF05-4, *B. uniformis* AF21-53, *B. uniformis* AF26-10BH and *B. uniformis* strain AM43-9), the homolog of the *B. plebieus* β-agarase (GH86, Bacple_01694) appeared to have recombined with a DNA fragment composed of two genes predicted to encode metabolic enzymes: 2-dehydro-3-deoxygluconokinase and 2-dehydro-3-deoxyphosphogluconate aldolase (S5 Fig). For these last four strains, a deletion of the genes encoding the GH50 (Bacple_01683) and GH105|GH154 (Bacple_01684) located in *PUL-PorC* was also observed, highlighting the remodeling of the PUL by insertion and deletion of DNA segments (S5 Fig). Note that parts of *PUL-PorA* and *PUL-PorC* were incompletely sequenced in *B. plebeius* strain AM09-36 and *B. eggerthii* strain AM42-16.

To explore further the phylogenetic diversity of *PUL-PorB*, we scanned the whole catalog of Almeida et al [34], including not only the previously mentioned 10,969 gut bacterial isolates, but also 278,263 gut bacterial metagenomes-assembled genomes (MAGs) coming from 13,587 worldwide individuals, which we completed with 29,082 MAGs coming from 842 individuals from under-represented Asian countries (Japan, India, Korea) [35]. We used BLASTn to identify homologs of the six known genes from *PUL-PorB* and found overall 245 strains having a full *PUL-PorB* sequence, which were reduced to 130 non-redundant ones after removing strains from the same bacterial species having 100% identity (S3 Table). This analysis revealed eight additional *Phocaeicola*/*Bacteroides* species that acquired the porphyran PUL, seven of which had never been described before (*B. caccae*, *B. cellulosilyticus*, *B. coprocola*, *B. finegoldii, B. ilei, B. massiliensis* and *B. stercorirosoris*). Interestingly, *PUL-PorB* was absent in other common *Bacteroides* sister species from the human gut such as *B. intestinalis*. We also identified it in two species of *Parabacteroides* (*P. johnsonii* and *P. merdae*), as well as in two new genera: *Tyzzerella sp*. and *Holdemanella sp*. which belong to the Bacillota phylum. Overall, the porphyran PUL is thus now known to be present in 19 *Bacteroides* or *Parabacteroides* species, and three genera from the Bacillota phylum (*Faecalicatena contorta*, identified in [13], *Tyzzerella* and *Holdemanella* identified here).

In terms of the prevalence of these different *PUL-PorB*-carrying bacterial species, we observed that four species were the most frequent, with quite equal contributions (*B. plebeius*, *B. uniformis*, *B. vulgatus*, and *B. dorei,* corresponding each to 15–19% of all positive strains), while others were more anecdotal (<3%), outside of *B. coprocola* being intermediate (7%) (S6A Fig). Of note, outside of *B. plebeius*, none of the most frequent species had been identified before.

### Genetic diversity of *PUL-PorB*

The neighbor-joining tree of these 130 non-redundant strains, calculated with the six concatenated protein sequences of *PUL-PorB* (Fig 5A), revealed four clades with high bootstrap values, allowing us to classify the strains in four groups: GI, GII, GIII and GIIIrec. The percentage of identity of each *PUL-PorB* protein was compared with the corresponding sequence of *B. plebeius* DSM 17135 (S3 Table). In the group GI, which includes the *B. plebeius* DSM 17135 reference, most protein sequences presented 99.5−100% identity with the reference. Similarly, for the members of the group GII, the protein sequences were 99−100% identical to each other, but presented 96−100% identity with the *B. plebeius* reference. At the root of the GI clade (Outgroup GI), the concatenated sequences of *PUL-PorB* of 5 strains presented lower identity (98−100%), reflecting some recombination events. For example, the isolated *B. plebeius* strain AM49-7BH suggested that its divergence with GI members resulted from a recombination event that occurred between SusD, SusC and GH2 genes from GI and Sulf and GH29 genes more related to GII. The recombination site was identified in the gene coding for GH16 (S7 Fig).

The clades GIII encompasses members which presented 92–98% identity with the *B. plebeius* reference DSM 17135. The *PUL-PorB* sequence of the GIIIrec members seems to result from a recombination event between GI and GIII (S3 Table), since the SusD (bacple_01704), SusC (bacple_01705) and GH2 (bacple_01706) sequences presented 99–100% identity with GI, while the S1_11 sulfatase (bacple_01701) and GH29 (bacple_01702) were nearly identical to GIII sequences. Again, the recombination site was identified in the gene coding for GH16 (S7 Fig).

Investigating whether there is any correlation between the taxonomic identity of the bacterial species carrying the *PUL-PorB* and these clades of *PUL-PorB*, we observed that GI is mostly carried by *B. uniformis* and *B. dorei*, while GII is mostly carried by *B. vulgatus*, *B. dorei* and *B. uniformis*, and GIII/GIIIrec is predominantly carried by *B. plebeius* and *B. coprocola* (S6B Fig). It thus appears that the three different *PUL-PorB* groups are not carried by the same set of bacterial species (chi-squared test, p-val < 2.2e-16). Of note, *Bacillota* are present in the three groups but represent a very minor proportion of bacteria carrying the *PUL-porB*.

### Geographic distribution of *PUL-PorB* in assembled metagenomes

We then explored the worldwide geographic prevalence of the porphyran degradation system in diverse human gut metagenomes using the catalog from Almeida et al [34] combined with the dataset from Kim et al [35] including 842 individuals from under-represented Asian countries (Japan, India and Korea). This corresponds in total to 14,429 individuals from 32 countries (13 from Europe, 9 from Asia, 6 from America, 2 from Oceania and 2 from Africa). We found that 26% of individuals were positive both in Japan and Korea, while only 3% of individuals were positive in China. All other countries had no hits or prevalence lower than 1% (from higher to lower prevalence: 1/110 positive individual in Mongolia, 1/238 in Fiji, 2/759 in Sweden, 7/3036 in USA, and 2/954 in Israel), as also shown in [13], even though they did not provide quantitative estimates.

Investigating further the prevalence across China by manually downloading and scanning individual metagenomic projects (S4 Table), given that the catalog from Almeida et al [34] only provides a geographic resolution by country, we realized that the prevalence obtained in the original Chinese projects were much higher than in Almeida et al (in average 36% versus 3% [34]). This seems to be because the catalog from Almeida et al [34] applied very stringent filters. Indeed, to have a high-quality catalog, they removed the contigs that had less than 50% completeness and a quality score less than 50. As a consequence, the number of metagenomes per individual are about ten times lower than in the original projects. The dataset compiled by Almeida et al [34] is thus useful to identify which populations carry or not the porphyran PUL, but is not suited to obtain accurate prevalence. We thus manually scanned a total of 20 downloaded projects covering 3,556 biosamples from 10 countries for which gut metagenomes were obtained and assembled (S4 Table). We found that in East Asia, 29% of individuals were positive (585 out of the 2,016 tested) – corresponding to 33% of the Chinese individuals, 25% of the Japanese individuals and 26% of the Korean individuals, while we found only six positive individuals among the 1237 North American gut metagenomes (0.5% of the individuals) and none among the other geographic areas, including 117 individuals from South Asia (India, Bangladesh), 36 from South America (Peru), 139 from Africa (Tanzania, Madagascar) and 11 from Europe (Italy) (S3 Table).

Overall, consistently with previous investigations, the occurrence of algal polysaccharides degradation systems seems to be mostly restricted to East Asia (Japan, China, Korea) and not be present at appreciable frequencies in other Asian countries (Kazakhstan, Mongolia, India, Bangladesh, Singapore) or elsewhere in the world.

The 591 positive individuals from these original 20 metagenome projects were then characterized as belonging to GI, GII, GIII and/or GIIIrec based on their *PUL-PorB* gene sequence (S5 Table). For 95.4% of the 585 positive East Asian individuals, the genes detected were attributed to a single group; however, few individuals (4.6%) presented *PUL-PorB* genes that belonged to two different groups. The individuals were then grouped by geographic location (Fig 5B). In order to obtain large sets of individuals, we distinguished populations living in North-East China (Hangzhou), South-East China (Shenzen and Hong-Kong), Korea and Japan. In both set of Chinese individuals, the group GI was present in more than half of the population (57% and 55%, respectively) followed by the group GII (33% and 39%, respectively). In contrast, in Japan, GII was the major group, found in twice higher proportion than GI (55% and 28%, respectively). GIII and GIIIrec were minor groups in these populations, with GIII found in higher proportion in Japan (16%) and GIIIrec in higher proportion in China (14%, 4% and 0% in Hangzhou, Shenzen and Hong-Kong, respectively). In Korea, even though much fewer individuals are analyzed (n = 23), the picture seems to be different from both China and Japan, with GIII being the major group (43%) and GI, GII being at similar frequencies (22% and 26%, respectively).

### Analysis of short read datasets

The analyses of the assembled metagenomes revealed that 20% to 30% of individuals carry the *PUL-PorB* in East Asia (Japan, China, Korea), but that it is mostly absent elsewhere (<1%). In addition, 95.2% of the individuals carry only one version – one group – of the *PUL-PorB*. These observations may however include some bias inherent to the bioinformatics processes involved in the building up of the contigs. Therefore, to analyze the data devoid of such biases, we have selected a 54 nucleotides sequence allowing to probe the different *PUL-PorB* groups directly on raw sequencing data (short reads). This allowed us to explore additional metagenomic datasets which were not assembled, thus expending the number of sampling sites, as well as the number individuals tested, with now a total of 4,617 individuals (S4 Table).

As observed previously, we confirmed by this complementary approach that *PUL-PorB* is not found at appreciable frequencies outside East Asian coastal populations, i.e., in America, Africa or other parts of Asia (Mongolia, India, Malaysia), where the prevalence is null to extremely low (3% at most in Mongolia) (S4 Table, Fig 6). The only exception, however, is in Malaysia where the prevalence in Kuala Lumpur was found to be 16% (but 1% in rural Malaysia), likely reflecting migration fluxes from China. Across Asia, the prevalence obtained are quite higher than the ones obtained with the assembled datasets (50% in China, 72% in Japan and 89% in Korea), with also a certain variance across datasets in Japan (15–94%) and in China (20–70%).

Because of the variability in sequencing depths across datasets, we tested whether the prevalence of *PUL-PorB* could be influenced by coverage (with the idea that more false negatives could be observed in datasets with too little coverage). However, we did not observe a significant effect of coverage on *PUL-PorB* prevalence (Pearson correlation coefficient r = 0.14, p-val = 0.499).

We further found that there is no effect of age (chi-squared test, p-val = 0.086), even though metadata on age was available only for two Chinese projects (PRJNA422434 and PRJNA356225); we did not find a significant effect of sex either in the Shenzen dataset (PRJNA422434: chi-squared test, p-val = 0.158).

We then estimated the proportion of bacteria carrying *PUL-PorB* for each positive individual by dividing the number of reads blasting against *PUL-PorB* by the total number of reads. We found that it varies between 10^-11^ to 10^-7^ between individuals (with absolute counts being between 1 and 3099), with a median of 10^-9^. Interestingly, Chinese have 3–4 times lower abundances of *PUL-PorB* than Koreans or Japanese (0.7x10^-9^ versus 2.6x10^-9^ and 3.2x10^-9^, respectively, ANOVA p-val < 2.2e-16, S9 Fig).

Because of the variability in sequencing depths across individuals, we then excluded individuals with too few (less than 10) reads to characterize their *PUL-PorB* genetic diversity. Among 2138 positive individuals, we thus retained the 1171 individuals having at least 10 reads. The histograms presented in S10 Fig show the number of hits for these individuals and their attribution to the GI, GII, GIII and GIIIrec groups. We found that 60% of individuals carried short reads attributed to a single group, and an additional 21% of individuals had a dominant group, defined as having a prevalence of the major group above 80%. Thus, we estimated that overall, 81% of individuals carried only one or a dominant group. Quite notably, more individuals carried a single or dominant group in China and Japan (84%) as compared to Korea (53%), where half of the individuals carried at least two groups, none of which exceeding 80% in frequency. To verify this is not due to the about five times higher coverage in Korean samples, we looked at the 52 Korean individuals with less than 100 reads (corresponding to individuals with a median number of positive reads of 42, thus quite similar to the median in Japanese of 47 and that in Chinese of 29), and we found a similarly low number of individuals with one or a dominant group (39%) in this subset.

Looking at the prevalence of individuals being positive for each group, we confirmed that GI were predominant in Chinese populations (52% of individuals, versus 33% in Koreans and 32% in Japanese), while GII was most common in Japanese populations (62%, versus 53% in Koreans and 46% in Chinese) (Fig 7). Finally, the GIII and GIIIrec groups were more prevalent in Koreans (60%) as compared to Japanese (25%) and Chinese (14%).

We then tested whether there were significant differences in group prevalences across cities and countries. Including only categories with large enough numbers (more than 10 individuals overall), we found that the six populations were overall significantly different in their group prevalence (X-squared = 324.41, p-value < 2.2e-16) (Fig 7). This difference was mostly driven by significant differences between countries (all comparisons between countries: q-value < 1e-04), as well as between Hong-Kong and Hangzhou, and Hong-Kong and Shenzen/Guangzhou (q-value = 0.027 and 0.044, respectively). We then tested separately which group was significantly different in prevalence between populations and found that all were significantly different (proportion test, q-value < 5e-06), but GIII and GIIIrec presented the stronger difference (proportion test, q-value = 1.1e-15).

## Discussion

Biochemical and crystallographic investigations showed that the porphyran PUL encodes tailored enzymes dedicated to the complete depolymerisation of the polysaccharide, including the methylated fraction, giving galactopyranoside and 6-O-methyl-galactopyranoside as end-products. We showed that the 6-O-methyl-porphyranase (BpGH16C) presents an active site able to accommodate the methyl group, in contrast with the active sites of the previously investigated β-porphyranases. Similarly, the exo-6O-L-galactose porphyran sulfatase Bp S1_11 can accommodate methylated oligosaccharides in its active site. The demethylation of 6-O-methyl-galactopyranoside was located in agarose PUL of several aerobic bacteria belonging to the Pseudomonadota, Bacteroidota and Planctomycetota phyla. The reaction was obtained with specific monooxygenase enzyme system, including ferredoxin, ferredoxin reductase and P450 monooxygenase [36]. Although, demethylation of galactose residue in anaerobic bacteria has not been elucidated yet and no encoding gene surrounding the porphyran utilization loci could be suspected, one can hypothesized that demethylation probably occurs in anaerobic bacteria by a mechanism to be discovered. Nevertheless, the degradation of the methylated fraction released, at least, the non-methylated D-galactose residue of the methylated-porphyranobiose moiety.

Thanks to a large catalog of assembled gut bacterial genomes [34,35] including both isolated strains and metagenome-assembled genomes (MAGs), we were able to interrogate an important number of sequences (about 300,000) and show that more than a hundred different strains carry the porphyran PUL, corresponding to 12 *Bacteroides* species as well as two other genera from the Bacillota phylum (*Tyzzerella sp*. and *Holdemanella sp*.). The gene organization of the porphyran PUL is furthermore quite conserved, with a minority of rearrangements events (i.e., loss or duplication of genes). This possibly indicates a unique common ancestor of these PUL from the environment, and then the occurrence of multiple transfers of this system across closely related gut bacterial species by horizontal gene transfer. Importantly, within a given bacterial species, strains carrying or not the porphyran PUL coexist. As a consequence, the taxonomic composition of an individual is not a good predictor of its metabolic capacity.

While our study aims to explore the phylogenetic distribution, diversity, and geographic prevalence of close homologs to the porphyran PUL originally discovered in *B. plebeius* and does not ambition to describe the whole diversity of other potential porphyran PULs, we did not use very strict BLAST parameters to allow for some diversity to be uncovered. Indeed, we used a threshold of 80% identity and 95% coverage, which resulted, for Bacple_01703 encoding the methyl-porphyranase BpGH16C, in the exclusion of 7,1% of hits, with all remaining hits having more than 80% identity but between 6–91% of query coverage.

As already shown, these transfers seem to happen preferentially between phylogenetically closely related bacteria [37,38], as the strains carrying the PUL were mostly found in the genera *Bacteroides*. While it would be interesting to know more about the timing of the original transfer of the porphyran PUL from the marine to the gut bacteria, the environmental bacteria from which the transfer occurred has not been identified (and does not seem to be present in current databases). Indeed, searching marine bacterial genomes and metagenomic data (e.g., Tara Ocean) failed to identify a porphyran PUL with similar genes organization or genes with high homologies to those observed in the human gut. Therefore, it is hard to establish when and how the porphyran PUL has evolved from its marine ancestor.

Because harvesting, drying and cooking *Porphyra sp*. were conserved practices in numerous populations living not only along the Pacific but also in Europe, and because these ancestral traditions seem to follow the dispersion line of *Homo sapiens* from Asia to the South of the American continent, we wondered if the porphyran degradation system may be present in a larger set of populations than the Chinese and Japanese where it was previously identified. We again took advantage of the large catalog of MAGs from Almeida et al [34], which we completed with another dataset from Japanese, Korean and Indian populations [35] to evaluate the geographic prevalence of the porphyran PUL degradation system. This allowed us to precisely define the distribution area of this system, which appears to be limited to coastal East Asian populations (Japan, China, Korea), as well as some continental Chinese populations and few individuals from urban Malaysia (potentially due to Chinese immigration). However, it is not found in neighboring countries such as Mongolia, rural Malaysia or India (nor Peru or Italy), suggesting, as expected, that strains carrying the PUL are not selectively retained in populations that do not include algae in their diet. Further studies including coastal countries in South America would be interesting to assess whether the PUL is present there.

While the catalog from Almeida and co-workers [34], a compilation of many datasets covering more than 13,000 individuals, was very useful to be able to scan the gut microbiome at a large geographic scale, the prevalence obtained on that catalog seems to be biased downwards (4% in China versus 33% on the same manually downloaded datasets). Indeed, for the assembled genomes to be of high quality, stringent quality filters were applied to the catalog. We thus could not exploit that catalog to obtain reliable prevalence data, which we instead estimated in the original projects. This analysis revealed a prevalence of the porphyran PUL of about 30% in East Asia. This number is itself quite different from the one obtained when analyzing directly the short-read data (unassembled), where we observed a prevalence of 50% in China, 72% in Japan and 89% in Korea. The most plausible explanation is that this genomic region is quite hard to assemble, because it is found on different bacterial backgrounds. Bacterial long-reads data, which start to emerge to look at bacterial structural variation [39], might help us better understand this discrepancy between the short-reads data and the assembled datasets. In any case, there seems to be a higher proportion of individuals carrying the PUL in Korea and Japan than in China, as well as three times more reads blasting against the PUL (reflecting the abundance of PUL-positive bacterial strains) in positive individuals from Korea and Japan compared to China. This might indicate that PUL-positive bacterial strains thrive more in the gut of Koreans and Japanese, probably because of a higher amount of porphyran in their diet, and thus, their gut. Some discrepancies remain to be explained, though, notably the large difference between the prevalence of the porphyran PUL in two datasets from Japan, one from Kanazawa (15%) and one from Tokyo (76%).

While we did not detect a significant effect of age on the prevalence of PUL-carrying bacteria when merging the data from the only two datasets with age information, we still identified a trend toward a lower prevalence in individuals over 40 years old, which was significant in the dataset from Shenzen alone (PRJNA422434, p-val = 0.023). This effect deserves to be further investigated to see whether it is replicated in other cohorts and to test whether this relates to different dietary habits between younger and older persons.

Investigation of the genetic diversity within *PUL-PorB* showed a certain amount of genetic structure across individuals which allowed us to define three clear clusters of sequences, as well as a recombinant group between two of them: GI, GII, GIII and GIIIrec. These groups are furthermore associated with somehow different bacterial species, and are found in different proportions across countries. Notably, GI was found in higher frequency in China, GII in Japan and GIII in Korea. Such significant differences in frequency between countries are what we expect to observe in a model where all populations are not entirely inter-connected. Conversely, the higher similarity between different populations in China might indicate that these populations are more connected in term of migration, and thus in term of exchanges of bacteria between individuals by horizontal transmission. However, even though differences in frequency are observed, all *PUL-PorB* groups (GI, GII, GIII, GIIIrec) are observed in all populations, showing that these strains do circulate at a quite large geographical scale. In general, this system is a very unique one, where we can track the migration of individuals through the diffusion of the PUL. It would be interesting to use it as a model to estimate the rate of horizontal transmission of gut bacteria between individuals at greater or lesser geographic and cultural distances.

Interestingly, analyses of both the MAGs and the short reads revealed that most individuals (80%) carry a single PUL variant, or a dominating one (even though less so in Korea – 53% of individuals), giving the impression of an apparent haploidy of the PUL. This observation has also been made for 80% of gut bacterial species, where one strain is dominant [40], so this might be a property of how bacteria establish and evolve in this peculiar environment.

In conclusion, the porphyran PUL encodes a portfolio of enzymes catalyzing the complete depolymerization of the methylated and unmethylated fractions of the polysaccharide without the help of external enzymes. While the architecture of the human gut microbiota across populations has mostly been studied and functionally interpreted at the genera or species level, we see here with the example of the porphyran degradation system that the important functional information is at the strain, or even the genetic level. Indeed, the PUL system is carried by various species and strains of the *Bacteroides* genus, so the inference of this metabolic capacity based on taxonomy only would not be possible. In this study, we demonstrated that geographically distant human populations (at the scale of East Asia) present different prevalence of PUL-carrying bacteria, and among positive individuals, these countries have similar groups, but at significantly different frequencies. Overall, the current structure of the investigated populations is likely the result of lateral transfer, recombination, as well as migration events, which reflect an evolution history of the gut microbiota and therefore, of its host.

## Materials and methods

### Purification of porphyran

6 g of dried *Porphyra columbina* were suspended in 120 ml de distilled H_2_O and autoclaved for 30 min at 120°C. After 14 h at room temperature, the suspension was centrifuged at 8000 rpm during 30 min at 4°C. The supernatant was added to 120 ml of pure ethanol (50% v/v EtOH final concentration) and the solution was maintained at 4°C for 2h. After centrifugation (8000 rpm, 30 min, 4°C), the porphyran, present in the supernatant, was precipitated by addition of 130 ml of pure ethanol (67% v/v EtOH final concentration). After centrifugation the porphyran pellet was dissolved in distilled water, and dialysis for 3 days against distilled water using dialysis membrane with a cut-off 1000 Da (pre-wetted Spectra/Por® 6 dialysis tubing – Spectrum labs). The polysaccharide was lyophilized and the purity was verified by ^1^H-NMR. The yield of purification was about 15–20% w/w dried algae.

### Heterologous expression of *Bacteroides plebeius* DSM 17135 PUL-PorB enzymes

The genes encoding the predicted glycoside hydrolases (BpGH29, Bacple_01702; BpGH16C, Bacple_01703; BpGH2C, Bacple_01706) and sulfatase (BpS1_11, Bacple_01701) from *B. plebeius* DSM 17135 were cloned using genomic DNA as template in the pET-28 or pFO4 expression plasmid [41] without their signal peptides identified with SignalP [42]. The expression strains harboring the recombinant expression plasmids were grown in Luria Bertani (LB) medium supplemented with 50 µg/ml kanamycin (pET28a plasmid) or 100 µg/ml ampicillin (pFO4 plasmid) until the OD_600nm_ reached 0.6 in a shaking incubator working at 180 rpm and 37°C. After the addition of isopropyl-β-D-thiogalactopyranoside (IPTG), the temperature was cooled down at 20°C and maintained overnight.

Cultures (200 mL) were centrifuged at 6000 g for 15 min and the bacterial pellet was suspended in 10 ml of buffer A (20 mM Tris pH 8, 500 mM NaCl, 20 mM Imidazole). The cells were lysed using a cell disrupter (Constant system Ltd). Insoluble fractions were removed by centrifugation at 20000 g during 30 min at 4°C and the supernatant was loaded on a 1 mL HisTrap^TM^ HP column (GE Healthcare) connected to a NGC chromatography system (BioRad). The proteins were eluted with a imidazole gradient from 20 to 300 mM gradient of imidazole. Pure enzymes were obtained after a size exclusion chromatography using a HiLoad 16/600 Superdex 75 pg column in buffer B (10 mM Tris-HCl pH 8, 50 mM NaCl).

#### Enzymatic assays.

Enzymatic degradations were monitored by analytical gel permeation chromatography using Superdex S200 10/300 and Superdex peptide 10/300 (GE Healthcare) columns mounted in series and connected to a high-performance liquid chromatography (HPLC) Ultimate 3000 system (Thermo Fisher). The injection volume was 20 µL and the elution was performed at 0.4 mL.min-1 in 0.1 M NaCl. Oligosaccharides were detected by differential refractometry (Iota 2 differential refractive index detector, Precision Instruments).

The oligosaccharide products were purified by semi-preparative gel permeation chromatography using three HiLoad® 26/600 Superdex® 30 pg (GE Healthcare) columns mounted in series and connected to a semi-preparative size-exclusion chromatography system which consisted of a Knauer pump (pump model 100), a refractive detector (iota2 Precision instrument) and a fraction collector (Foxy R1) mounted in series. The elution was conducted at a flow rate of 1.2 mL.min^-1^ at room temperature using 100 mM (NH_4_)_2_CO_3_ as eluent. The collected fractions were freeze-dried prior NMR and mass spectrometry analyses.

Samples were exchanged twice with D_2_O and were transferred to a 5 mm NMR tube. ^1^H NMR spectra were recorded at 323 K using an Advance III 400 MHz spectrometer (Bruker). Chemical shifts are expressed in ppm in reference to water. The HOD signal was not suppressed.

#### NMR.

^1^H NMR spectra were recorded with a Bruker Advance 400 spectrometer operating at a frequency of 400.13 MHz. Samples (2% w/v) were solubilized in D_2_O at a temperature of 293 K for the oligosaccharides and 353 K for the polysaccharide. Residual signal of the solvent was used as internal standard: HOD at 4.85 ppm at 293 K and 4.35 ppm at 343 K. Proton spectra were recorded with a 4006 Hz spectral width, 32,768 data points, 4.089 s acquisition times, 0.1 s relaxation delays and 16 scans.

#### Crystallization, data collection and structure solutions.

The homogenous fractions of proteins obtained from gel permeation chromatography were concentrated and crystallization experiments were attempted. Initial crystals were obtained by screening against wide range of commercial screens and the hits were optimized by hanging drop diffusion method. The drop containing 1 μl of protein and 1 μl of reservoir solution was incubated over 1 ml of reservoir solution and crystal growth was monitored regularly. The conditions of the best diffracting crystals of the three proteins were listed: BpS1_11 (Bacple_01701) was crystallized at 23 mg/ml in 40% Peg200, 0.1 M Sodium Citrate buffer pH 5.5 and 30% MPD; BpGH29 (Bacple_01702) was crystallized at 18 mg/ml in 20% Peg 8K and 0.1 M KH_2_PO_4_; BpGH16C (Bacple_01703) was crystallized at 34 mg/ml in 16% Peg 8K, 0.1 M Hepes pH 7.5 and 0.2M Calcium acetate. For diffraction experiments, the crystals were cryo protected in reservoir solution containing 20% MPD (BpS1_11), 25% glycerol (BpGH29), 30% ethylene glycol (BpGH16C) and flash frozen in liquid nitrogen. The diffraction data of all the crystals were collected at the 08ID beamline at the Canadian Light Source [43].

The X-ray diffraction data were processed using XDS [44]. The structures were solved by molecular replacement using the program Phaser in Phenix package [45]. For BpS1_11, the structure solution was obtained by molecular replacement using the search model an arylsulfatase from *Pseudomonas aeruginosa* (PDB ID 1HDH) [46]. For BpGH29, the structure solution was obtained using the α-L-fucosidase from *Fusarium graminearum* as a search model (PDB ID 4NI3) [47]. BpGH16C was solved using porphyranase B from *Zobellia galactanivorans* (PDB ID 3JUU) [9] as a model. All the structures were refined with the Phenix software [48] and manual rebuilding and solvent placement was conducted with the COOT program [49]. The stereochemistry of all the models were validated with MolProbity [50].

To obtain the complex structure, putative active site mutants of BpS1_11 (BpS1_11(H214N)) and BpGH16C (BpGH16C(E145L)) were made using the Quickchange site-directed mutagenesis protocol and using KOD polymerase. Briefly, the plasmid containing the gene of interest was amplified with the primer pairs carrying the mutation using KOD polymerase. After PCR, the template plasmid was digested with 1 µl of DpnI enzyme for an hour at 37°C. Five µl of PCR product was then transformed into 50µl of chemically competent *E. coli* DH5α cells. The clones carrying the desired mutation were confirmed by sequencing and proceeded for crystallization experiments.

Based on the structural comparison with other sulfatases, the His 214 residue in BpS1_11 was mutated to Asn (H214N). The mutant protein BpS1_11(H214N) was expressed and purified following the same protocol used for wild type enzyme. Purified Bacple_01701(H214N) was crystallized from solution containing 40% PEG 200, 0.1M sodium citrate buffer pH 5.5 and 30% MPD. The putative active site mutant of BpGH16C(E145L) was purified following the same protocol used for wild type enzyme. BpGH16C(E145L) was crystallized from solution containing 20% PEG 8K, 0.1 M Hepes pH 7.5 and 0.2 M calcium acetate. BpS1_11(H214N) and BpGH16C(E145L) crystals were soaked in the tetrasaccharide solution for an hour before the diffraction experiments. The soaked crystals were flash frozen in liquid nitrogen and diffraction data was collected at the 08ID beamline in the Canadian light source [43]. The diffraction data was processed with XDS [44]. One round of rigid body refinement was carried out using Phenix refinement program [48]. Manual rebuilding and substrate placement were done using coot [49]. The geometry was validated using MolProbity program [50].

#### Human gut analyses.

First, we downloaded and explored the 10,969 genomes of bacteria isolated from the human gut from the unified catalog of Almeida et al. [34]. Bacterial strains harboring the porphyran PUL were identified using BLASTn with the entire *PUL-PorA* (17,980 bp), *PUL-PorB* (12,888 bp) and *PUL-PorC* (19,198 bp) sequences, respectively, against the reference genome of *B. plebeius*. We retained isolates as positive if they had a percentage of identity equal or above 80%, including partial hits (no filtering on coverage), in order to capture phylogenetically diverse species. We kept all strains that had at least one hit in one of the three *PUL-Por* (whether A, B or C). For the rest of the manuscript, we assessed only *PUL-porB* as it was the most conserved part of the *PUL-por*.

Then, we downloaded the whole catalog of Almeida et al [34], including not only the previously mentioned 10,969 gut bacterial isolates, but also 278,263 gut bacterial metagenomes-assembled genomes (MAGs) coming from 13,587 worldwide individuals, which we completed with 29,082 MAGs coming from 842 individuals from under-represented Asian countries (Japan, India, Korea) [35]. Using BLASTn, we retained hits with at least 80% identity and at least 95% coverage to each of the six genes of *PUL-porB* (bacple_01701 to bacple_01706) and considered individuals/strains as positive if they carried all six fully sequenced gene of *PUL-PorB*. In order to verify whether these sequences were complete and functional, we further used BLASTp and aligned the protein sequences (concatenated or not) using Muscle. We then manually excluded truncated proteins or sequences containing a STOP codon.

Phylogenetic trees were built using concatenated sequences with MEGA6 [51], allowing to overall distinguish GI, GII, GIII and GIIIrec groups. However, to validate our classification, we also obtained the phylogenetic trees for each protein separately. Indeed, to identify GIIIrec, we required to observe some proteins of the same contig matching GI while others matched GIII. When only part of the proteins was available, we considered the major group (i.e., GI or GIII) as the most probable one, creating a slight bias downward for GIIIrec. When multiple contigs in the same individual were found to be positive and they corresponded to different groups, we considered the individual as multi-groups (e.g., GI + GII). When we could not assign a contig to one of the 4 groups defined earlier, we considered the individual to be unresolved (outgroup).

To be able to use more diverse non-assembled datasets, we further designed a 54 nucleotides sequence allowing to probe the different *PUL-PorB* groups directly on raw sequencing data (short reads). The probe is part of the Bacple_01703 (GH16) gene encoding the methyl-porphyranase, which presents distinct mutations specific to the GI, GII, GIII and GIIIrec groups (S8 Fig). Analyses of short reads datasets were conducted with BLASTn using these four 54 nt probes, requiring hits to have 100% identity and 100% coverage to the 54nt probes. The probes were used to search at Sequence Read Archive (SRA) data at NCBI. To obtain an estimation of the sensitivity and the specificity of these probes, we compared the hits obtained in the catalogue of Almeida et al [34] with the four probes to the ones obtained with the six *PUL-porB* genes. Among 289,232 MAGs or isolates, 155 sequences were positive with both approaches, while only 3 were not found with the probes, and 30 were found only with the probes. This gives us a sensitivity of 98% and a specificity of 100%, validating the use of these probes to assess the prevalence of *PUL-porB* in unassembled data.

## Supporting information

S1 Fig **A**) Chemical structure of the repetition moieties of porphyran. The sulfated disaccharides – porphyranobioses – can present methyl group at the position 6 of the D-galactose giving methylated and none-methylated porphyran components. Agarobiose units, obtained by desulfation/cyclization of the L-galactose residue during the biosynthesis, are methylated accordingly. **B**) Organization of the B. plebieus porphyran PUL. The PUL is divided in three segments (*PUL-PorA*, -*Por B* and -**PorC**) based on previous transcriptomic analyses. The cluster Bacple_01692 to Bacple_01699 genes – named *PUL-PorA*– was moderately up-regulated when *B. plebieus* was grown in the presence of porphyran. This contrasts with the two neighboring clusters of genes: Bacple_01668 to Bacple_01689 (*PUL-PorC*) and Bacple_01700 to Bacple_01706 (*PUL-PorB*), which were highly up-regulated (10-fold more than PUL-PorA) [9]. The enzyme functions were determined in (1) Hehemann et al., 2012, (2) Giles et al., 2017, (3) Rodd et al., 2022 and (4) this study.
(EPS)

S2 Fig Structural alignment of the *B. plebieus* DSM 17135 BpGH16C 6-O- methyl-β-porphyranase compared with the other characterized 6-O-methyl-β-porphyranases (*P. atlantica* T6c, Patl_0824; *W. fucanilytica*, AXE80_06940) and characterized β-porphyranases (*B. uniformis* NP1, BuGH16A; *P. atlantica* T6c, Patl_0805; *B. plebeius* DSM 17135, BpGH16B). The blue circle indicates the serine/threonine amino acids observed in the active site of the β-porphyranases which are replaced by a glycine amino acid in 6-O-methyl-β-porphyranases allowing to accommodate the methyl group.
(EPS)

S3 Fig ^1^H NMR recorded before and after incubation with the sulfatase BpS1_11 on purified disaccharides end-products of the BpGH16C. The occurrence of the methyl group located at the position 6 of the D-galactose didn’t hinder the removal of the sulfate ester group of the L-galactose residue located at the non-reducing end.
(EPS)

S4 Fig **A**) Structural details of Bacple_01701. Schematic representation of Bacple_01701 shown in cartoon representation and colored from N to C terminus. Calcium ion is shown as green sphere. **B**) Conserved active site residues from S1 family sulfatases. The residues are shown in stick representation in the order Bacple_01701/ 5G2V/1HDH/6BIA. **C**) Surface representation of H214 mutant with the tetrasaccharide, showing pocket like architecture of active site. The 6S sulfate subsite is labelled as S. **D**) Complex structure of H214N mutant. The electron density (2fo-fc) map for the sugar contoured at 1.5σ. Sugars numbered from 0 to +3 and 6S sulfate subsite labelled as S. Residues at H bonding distance to the substrate are shown in stick representation. **E**) Structural comparison of H214 mutant with 1HDH. The residues involved in catalysis S83 (overlays with DDZ from 1HDH), H133, H214 (this is the mutated residue) are shown in stick representation.
(EPS)

S5 Fig Comparison of the two main gene organizations of the porphyran PUL observed in the set of isolated bacteria investigated highlighting deletion and recombination events. (EPS)

S6 Fig **A**) Taxonomic distribution of the species of 245 bacterial strains carrying the PUL-PorB. **B**) Taxonomic distribution in the set of bacteria carrying the *PUL-PorB* listed in the GI, GII and GIII/GIIIrec groups.
(EPS)

S7 Fig Recombination sites observed in the GH16 (Bacple_01703) of the *PUL-PorB.* The Outgroup GI (GIrec) was obtained by the recombination of one gene grouped in GI with one gene distantly related to the GII group (top). Recombination of genes of the groups GI/GIII produced GIIIrec (bottom).
(EPS)

S8 Fig Sequences of 54 nucleotides characteristic of the different groups of PUL-PorB used to Blastn SRA dataset. (EPS)

S9 Fig Relative abundance of short reads mapping to the 54-nucleotides probes specific to the PUL-PorB in positive individuals across Chinese, Japanese and Koreans projects. (EPS)

S10 Fig The 54 nucleotides sequence characteristic of different group of porphyran PUL were Blastn against the short read sequencing (SRA) of metagnomic data recorded on Japanese, Korean and Chinese populations. The number of detected short reads are indicated for each individuals and were grouped as a function of the geographic location of the sampling. Only individuals for which at least 10 reads were recorded are shown.
(EPS)

S1 Table ^1^H and ^13^C NMR chemical shifts of the oligo-porphyran series obtained after successive enzymatic reactions.
(PDF)

S2 Table List of the bacterial isolated strains which assembled genome contained genes of the porphyran degradation system. *Bacteroides* and *Phocaeicola* are basonyms. The name of the strains was reported as reported in databank.
(PDF)

S3 Table List of the 130 non-redundant bacterial strains containing the complete *PUL-PorB.* The percentage of identity of the protein sequences of the PUL-PorB were compared with those of the reference *B. plebeius* DSM 17135. The *PUL-PorB* group of each individuals/ isolate was determined based on the phylogenetic tree calculated on concatenated protein sequences as presented in Fig 4A.
(PDF)

S4 Table List of assembly projects probed with the genes encoding *PUL-PorB* and list of SRA projects probed with the 50 nucleotides probes. (PDF)

S5 Table List of the reconstructed *PUL-PorB* found in the assembled human gut metagenomes of Chineses, Japaneses and Korean populations. Only full length genes were reported in the list. The genes were classified in Group I, II, III or IIIrec based on their homology with those found in reference bacteria (Fig 4).
(PDF)
