# Supplementary material for: The porphyran degradation system is complete, phylogenetically and geographically diverse across the gut microbiota of East Asian populations
Source: PLoS One. 2025 Aug 1;20(8):e0329457. doi: 10.1371/journal.pone.0329457 (PMC12316285; doi:10.1371/journal.pone.0329457)
Supplement: S1 Table — 1H and 13C NMR chemical shifts of the oligo-porphyran series obtained after successive enzymatic reactions. (PDF) [file pone.0329457.s011.pdf]

S1 Table : ^{1}H and ^{13}C NMR chemical shifts of the oligo-porphyran series obtained after successive enzymatic reactions.

Sugar Residue			1	2	3	4	5	6 (6a,6b)	OMe
**DP2**									
**Gal**	6S-α-L-Gal*p*-(1→	^{1}H	5.30	3.89	4.01	4.11	4.36	4.27, 4.21	
		^{13}C	101.04/100.94 [a]	68.52	69.07	69.10	69.37	68.09,68.04	
**Galα**	→3)-α-D-Gal*p*	^{1}H	5.34	4.05	3.98	4.30	4.17	3.81, 3.78	
		^{13}C	92.25	67.45	78.06	69.31	70.74	61.38	
**Galβ**	→3)-β-D-Gal*p*	^{1}H	4.70	3.71	3.78	4.24	3.79	3.81, 3.78	
		^{13}C	96.21	71.10	80.96	68.65	75.28	61.22	
**Desulfated DP2**									
**Gal**	α-L-Gal*p*-(1→	^{1}H	5.31/5.30[a]	3.89	4.00	4.06	4.16	3.80	
		^{13}C	100.63	68.65	69.24	69.24	71.48	61.23	
**Galα**	→3)-α-D-Gal*p*	^{1}H	5.34	4.05	3.98	4.28	4.17	3.80	
		^{13}C	92.23	67.58	77.66	69.05	70.43	61.05	
**Galβ**	→3)-β-D-Gal*p*	^{1}H	4.70	3.72	3.78	4.22	3.78	3.80	
		^{13}C	96.23	71.14	80.94	68.39	74.98	60.87	
**DP2Me**									
**Gal**	6S-α-L-Gal*p*-(1→	^{1}H	5.30	3.90	4.01	4.11	4.36	4.27, 4.21	
		^{13}C	101.10/101.00 [a]	68.48	69.07	69.10	69.38	68.11/68.07	
**Galα**	→3)6OMe-α-D-Gal*p*	^{1}H	5.34	4.05	3.98	4.31	4.32	3.71	3.44
		^{13}C	92.22	67.30	78.02	69.51	68.63	71.98	58.18
**Galβ**	→3)-6OMe-β-D-Gal*p*	^{1}H	4.70	3.71	3.78	4.25	3.91	3.71	3.45
		^{13}C	96.13	70.94	80.92	68.88	73.26	71.86	58.35
**Desulfated DP2Me**									
**Gal**	α-L-Gal*p*-(1→	^{1}H	5.31/5.30[a]	3.90	3.99	4.05	4.15	3.80	
		^{13}C	100.66	68.63	69.25	69.25	71.47	61.24	
**Galα**	→3)6OMe-α-D-Gal*p*	^{1}H	5.34	4.04	3.98	4.26	4.30	3.68	3.44
		^{13}C	92.21	67.45	77.57	69.08	68.40	71.63	58.24
**Galβ**	→3)-6OMe-β-D-Gal*p*	^{1}H	4.70	3.72	3.78	4.21	3.90	3.68	3.45
		^{13}C	96.16	71.01	80.87	68.63	73.00	71.63	58.38
**DP3Me**									
**Gal**	6OMe β-D-Gal*p*-(1→	^{1}H	4.47	3.65	3.73	4.00	3.91	3.71	3.47
		^{13}C	103.07	70.94	72.40	b	73.07	71.45	58.38
**Gal**	→4)-6S-α-L-Gal*p*-(1→	^{1}H	5.34	3.93	4.02	4.35	4.46	4.35	
		^{13}C	101.07/100.96 [a]	b	b	78.43	69.53	67.17	
**Galα**	→3)6OMe-α-D-Gal*p*	^{1}H	5.34	4.05	4.01	4.30	4.35	3.71	3.44
		^{13}C	92.22	67.30	78.04	69.08	69.49	71.96	58.18
**Galβ**	→3)-6OMe-β-D-Gal*p*	^{1}H	4.70	3.72	3.80	4.24	3.91	3.71	3.45
		^{13}C	96.13	70.86	80.92	b	73.25	71.83	58.35

Sugar Residue			1	2	3	4	5	6 (6a,6b)	OMe
**DP4Me**									
**Gal**	6S-α-L-Gal*p*-(1→	^{1}H	5.30	3.88	4.00	4.11	4.35	4.27, 4.21	
		^{13}C	101.04	68.47	69.05	69.10	69.35	68.07	
**Gal**	→3)-6OMe β-D-Gal*p*-(1→	^{1}H	4.53	3.81	3.80	4.24	3.97	3.71	3.46
		^{13}C	103.12	70.26	80.50	68.72	73.10	71.61	58.37
**Gal**	→4)-6S-α-L-Gal*p*-(1→	^{1}H	5.34	3.94	4.02	4.36	4.46	4.36	
		^{13}C	101.08/100.95 [a]	c	c	79.05	69.50	67.39	
**Galα**	→3)6OMe-α-D-Gal*p*	^{1}H	5.34	4.05	4.00	4.30	4.35	3.71	3.44
		^{13}C	92.24	67.32	78.04	69.35	69.49	72.00	58.20
**Galβ**	→3)-6OMe-β-D-Gal*p*	^{1}H	4.70	3.72	3.80	4.24	3.91	3.71	3.45
		^{13}C	96.14	70.96	80.92	68.72	73.27	71.87	58.37
**Desulfated DP4Me**									
**Gal**	α-L-Gal*p*-(1→	^{1}H	5.33	3.88	4.00	4.05	4.13	3.80	
		^{13}C	100.57	68.60	69.05	69.24	71.46	61.24	
**Gal**	→3)-6OMe β-D-Gal*p*-(1→	^{1}H	4.53	3.82	3.83	4.21	3.96	3.71	3.46
		^{13}C	103.01	70.38	80.07	68.31	72.86	71.24	58.39
**Gal**	→4)-6S-α-L-Gal*p*-(1→	^{1}H	5.34	3.94	4.03	4.35	4.46	4.36	
		^{13}C	101.06/100.95 [a]	c	c	78.66	69.50	67.19	
**Galα**	→3)6OMe-α-D-Gal*p*	^{1}H	5.34	4.05	4.01	4.30	4.35	3.71	3.44
		^{13}C	92.22	67.32	78.05	69.24	69.49	71.96	58.18
**Galβ**	→3)-6OMe-β-D-Gal*p*	^{1}H	4.70	3.72	3.80	4.24	3.91	3.71	3.45
		^{13}C	96.12	70.94	80.92	68.85	73.25	71.84	58.35

. [a]: Gal→Galα / Gal→Galβ values, **b**=68.47-69.08 ppm, **c**=68.87;69.05 ppm, interchangeable values.
