# Supplementary material for: The porphyran degradation system is complete, phylogenetically and geographically diverse across the gut microbiota of East Asian populations
Source: PLoS One. 2025 Aug 1;20(8):e0329457. doi: 10.1371/journal.pone.0329457 (PMC12316285; doi:10.1371/journal.pone.0329457)
Supplement: S2 Table — Bacteroides and Phocaeicola are basonyms. The name of the strains was reported as reported in databank. (PDF) [file pone.0329457.s012.pdf]

Table S2: List of the bacterial isolated strains which assembled genome contained genes of the porphyran degradation system. *Bacteroides* and *Phocaeicola* are basonyms. The name of the strains was reported as reported in databank.

GenBank assembly	Assembly level	Biosamples	Strains
GCA_003466465.1	Scaffold	SAMN09736981	*Phocaeicola dorei* TM05-24
GCA_003467285.1	Scaffold	SAMN09736544	*Bacteroides eggerthii* AM42-16
GCA_003465275.1	Scaffold	SAMN09734245	*Bacteroides ovatus* AF14-13AC
GCA_000187895.1	Scaffold	SAMN00000028	*Bacteroides plebeius* DSM 17135
GCA_003472665.1	Scaffold	SAMN09734757	*Phocaeicola plebeius* AM09-36
GCA_003463985.1	Scaffold	SAMN09736616	*Phocaeicola plebeius* AM49-7BH
GCA_003465625.1	Scaffold	SAMN09734188	*Bacteroides stercoris* AF05-4
GCA_003468685.1	Scaffold	SAMN09736438	*Bacteroides stercoris* AM32-16LB
GCA_003468355.1	Scaffold	SAMN09736487	*Bacteroides stercoris* AM36-9BH
GCA_003460085.1	Scaffold	SAMN09734330	*Bacteroides uniformis* AF17-20
GCA_003459325.1	Scaffold	SAMN09734434	*Bacteroides uniformis* AF21-53
GCA_003468865.1	Scaffold	SAMN09736420	*Bacteroides uniformis* AM30-49
GCA_003466885.1	Scaffold	SAMN09736574	*Bacteroides uniformis* AM43-9
GCA_003437605.1	Scaffold	SAMN09736925	*Bacteroides uniformis* TF09-22
GCA_003458385.1	Scaffold	SAMN09734500	*Phocaeicola vulgatus* AF25-30LB
GCA_003474645.1	Scaffold	SAMN09734674	*Bacteroides xylanisolvens* AF38-2
GCA_003603275.1	Scaffold	SAMN09734277	*Bacteroides uniformis*. AF15-14LB
GCA_003603315.1	Scaffold	SAMN09734319	*Bacteroides uniformis*. AF16-7
GCA_003603065.1	Scaffold	SAMN09734409	*Bacteroides uniformis* AF20-13LB
GCA_003602995.1	Scaffold	SAMN09734502	*Bacteroides uniformis* AF25-38AC
GCA_003602945.1	Scaffold	SAMN09734509	*Bacteroides uniformis* AF26-10BH
GCA_003436255.1	Scaffold	SAMN09737012	*Bacteroides uniformis* D20 TM09-11
