# Supplementary material for: The porphyran degradation system is complete, phylogenetically and geographically diverse across the gut microbiota of East Asian populations
Source: PLoS One. 2025 Aug 1;20(8):e0329457. doi: 10.1371/journal.pone.0329457 (PMC12316285; doi:10.1371/journal.pone.0329457)
Supplement: S3 Table — The percentage of identity of the protein sequences of the PUL-PorB were compared with those of the reference B. plebeius DSM 17135. The PUL-PorB group of each individuals/ isolate was determined based on the phylogenetic tree calculated on concatenated protein sequences as presented in Fig 4A. (PDF) [file pone.0329457.s013.pdf]

Table S3: List of the 130 non-redundant bacterial strains containing the complete *PUL-PorB*. The percentage of identity of the protein sequences of the PUL-PorB were compared with those of the reference *B. plebeius* DSM 17135. The *PUL-PorB* group of each individuals / isolate was determined based on the phylogenetic tree calculated on concatenated protein sequences as presented in Figure 4A.

Ref catalog	Strain	MAG/ ISO	PUL-PorB Group	Bacple_01701	Bacple_01702	Bacple_01703	Bacple_01704	Bacple_01705	Bacple_01706
**GUT_GENOME096174**	**Bacteroides plebeius DSM 17135**	**ISO**	GI	100	100	100	100	100	100
GUT_GENOME154239	Bacteroides cellulosilyticus	MAG	GI	100	100	100	100	100	99.8
GUT_GENOME099338	Bacteroides coprocola	MAG	GI	100	100	100	100	100	100
KIJ_genome 1302	Bacteroides coprocola	MAG	GI	100	99.5	100	100	100	99.8
GUT_GENOME001074	Bacteroides dorei AF25-30LB	ISO	GI	100	100	100	100	100	99.8
GUT_GENOME032129	Bacteroides dorei	MAG	GI	100	100	100	100	99.9	99.8
GUT_GENOME239769	Bacteroides dorei TM05-24	ISO	GI	100	100	100	100	99.9	100
GUT_GENOME037305	Bacteroides dorei	MAG	GI	100	99.5	100	100	100	99.9
GUT_GENOME154531	Bacteroides dorei	MAG	GI	100	100	100	100	100	100
GUT_GENOME001766	Bacteroides eggerthii AM42-16	ISO	GI	100	100	100	100	100	99.8
KIJ_genome 5720	Bacteroides eggerthii	MAG	GI	100	99.5	100	100	100	99.9
GUT_GENOME155110	Bacteroides eggerthii	MAG	GI	100	100	100	100	100	100
KIJ_genome 13110	Bacteroides finegoldii	MAG	GI	100	99.5	100	100	99.9	99.9
GUT_GENOME098104	Bacteroides finegoldii	MAG	GI	100	99.5	100	100	100	99.9
GUT_GENOME249472	Bacteroides finegoldii	MAG	GI	100	100	100	100	100	100
GUT_GENOME101376	Bacteroides massiliensis	MAG	GI	100	99.5	100	100	100	99.9
GUT_GENOME000819	Bacteroides ovatus AF14-13AC	ISO	GI	100	100	100	100	100	99.9
GUT_GENOME030187	Bacteroides ovatus	MAG	GI	100	100	100	100	100	99.8
GUT_GENOME152759	Bacteroides ovatus	MAG	GI	100	99.5	100	100	100	99.9
KIJ_genome 25909	Bacteroides plebeius	MAG	GI	100	100	100	100	100	99.9
GUT_GENOME034154	Bacteroides plebeius	MAG	GI	100	99.5	100	100	100	99.8
GUT_GENOME250348	Bacteroides plebeius	MAG	GI	100	99.5	100	100	100	99.9
GUT_GENOME153158	Bacteroides plebeius	MAG	GI	100	100	100	100	99.9	100
GUT_GENOME001642	Bacteroides uniformis AM30-49	ISO	GI	100	100	100	100	100	100
GUT_GENOME000904	Bacteroides uniformis AF17-20	ISO	GI	100	99.5	100	100	100	99.9
KIJ_genome 736	Bacteroides uniformis	MAG	GI	100	99.5	100	100	99.9	99.9
GUT_GENOME155200	Bacteroides uniformis	MAG	GI	100	100	100	100	100	99.8
GUT_GENOME000851	Bacteroides uniformis AF15-14LB	ISO	GI	100	100	100	100	100	100
GUT_GENOME000893	Bacteroides uniformis AF16-7	ISO	GI	100	100	100	100	100	100
GUT_GENOME000983	Bacteroides uniformis AF20-13LB	ISO	GI	100	100	100	100	100	99.8
GUT_GENOME170222	Bacteroides uniformis	MAG	GI	100	99.3	100	100	100	99.9
GUT_GENOME239800	Bacteroides uniformis TM09-11	ISO	GI	100	100	100	100	100	100
GUT_GENOME001710	Bacteroides stercoris AM36-9BH	ISO	GI	100	100	100	100	100	100
KIJ_genome 10066	Bacteroides stercoris	MAG	GI	100	100	100	100	100	99.9
KIJ_genome 11367	Bacteroides vulgatus	MAG	GI	100	100	100	100	100	99.9
GUT_GENOME269537	Bacteroides xylanisolvens	MAG	GI	100	100	100	100	100	100
GUT_GENOME178534	Bacteroides xylanisolvens	MAG	GI	100	99.5	100	100	100	99.9
KIJ_genome 23951	Parabacteroides merdae	MAG	GI	100	100	100	100	99.9	100
GUT_GENOME027847	Bacteroides dorei	MAG	GI	100	99.3	100	100	100	99.8
KIJ_genome 12929	Tyzzerella sp000411335	MAG	GI	73.2	100	100	100	99.9	99.9
KIJ_genome 24121	Bacteroides coprocola	MAG	GI	98.3	100	100	99.4	99.8	99.6
GUT_GENOME178548	Bacteroides ovatus	MAG	GI	99.4	100	100	100	100	99.6
GUT_GENOME239713	Bacteroides uniformis TF09-22	ISO	GI	99.4	99	99.7	100	98.8	99.8
KIJ_genome 22831	Bacteroides plebeius	MAG	Outgroup GI	99.6	99.5	99.7	99.8	99.9	99.9
GUT_GENOME001839	Bacteroides plebeius AM49-7BH	ISO	Outgroup GI	99.8	99.3	99.7	99.8	99.9	99.9
KIJ_genome 28353	Bacteroides vulgatus	MAG	Outgroup GI	99.4	99	98	100	100	99.9
KIJ_genome 22636	Bacteroides uniformis	MAG	Outgroup GI	100	98.8	96.7	98.9	99.3	99.5
KIJ_genome 7975	Bacteroides plebeius	MAG	Outgroup GI	100	100	100	98.2	98.2	100
GUT_GENOME229839	Bacteroides stercorirosoris	MAG	Outgroup GII	99.8	99	96.7	97.4	98.2	99.5
GUT_GENOME034168	Bacteroides caccae	MAG	GII	99.8	99	96.7	97.4	98.2	98.7
GUT_GENOME149815	Bacteroides caccae	MAG	GII	99.4	99	96.7	97.6	98.2	98.7
KIJ_genome 15612	Bacteroides coprocola	MAG	GII	99.6	99	96.7	97.6	98.2	98.8
KIJ_genome 27310	Bacteroides coprocola	MAG	GII	99.4	99	96.7	97.4	98.2	98.7
KIJ_genome 20890	Bacteroides dorei	MAG	GII	99.8	98.8	96.7	97.4	98.2	98.8

KIJ_genome 2914	Bacteroides dorei	MAG	GII	99.8	99	96.7	97.6	98.1	98.8
KIJ_genome 22256	Bacteroides dorei	MAG	GII	99.4	99	96.3	97.6	98.2	98.7
GUT_GENOME151759	Bacteroides dorei	MAG	GII	99.4	99	96.3	97.6	98.2	98.7
GUT_GENOME027742	Bacteroides dorei	MAG	GII	99.4	99	96.7	97.4	98.2	98.7
GUT_GENOME027786	Bacteroides dorei	MAG	GII	99.8	99	96.7	97.4	98.2	98.8
GUT_GENOME028138	Bacteroides dorei	MAG	GII	99.8	98.8	96.7	97.4	98.2	98.8
GUT_GENOME046102	Bacteroides finegoldii	MAG	GII	99.4	99	96.7	97.6	98.2	98.7
GUT_GENOME080948	Bacteroides fragilis	MAG	GII	99.8	99	96.7	97.6	98.2	98.8
GUT_GENOME228260	Bacteroides fragilis	MAG	GII	99.8	99	96.3	97.6	98.2	98.7
KIJ_genome 14857	Bacteroides ilei	MAG	GII	99.8	99	96.7	97.4	98.2	98.8
KIJ_genome 16412	Bacteroides massiliensis	MAG	GII	99.8	99	96.7	97.4	98.2	98.8
GUT_GENOME148452	Bacteroides massiliensis	MAG	GII	99.4	99	96.3	97.6	98.2	98.7
KIJ_genome 17159	Bacteroides plebeius	MAG	GII	99.8	99	96.7	97.4	98.2	98.8
KIJ_genome 24998	Bacteroides plebeius	MAG	GII	99.8	99	96.7	98.1	98.2	98.8
KIJ_genome 157	Bacteroides plebeius	MAG	GII	99.4	99	96.7	97.4	98.2	98.7
KIJ_genome 26449	Bacteroides plebeius	MAG	GII	99.4	99	96.3	97.6	98.2	98.7
GUT_GENOME148536	Bacteroides plebeius	MAG	GII	99.4	99	96.7	97.4	98.2	98.7
GUT_GENOME001661	Bacteroides stercoris AM32-16LB	ISO	GII	99.4	99	96.7	97.4	98.2	98.7
GUT_GENOME027726	Bacteroides stercoris	MAG	GII	99.8	99	96.7	97.6	98.1	98.8
GUT_GENOME098019	Bacteroides stercoris	MAG	GII	99.8	99	96.7	97.6	98.2	98.8
GUT_GENOME246849	Bacteroides stercoris	MAG	GII	99.8	99	96.7	97.4	98.2	98.8
KIJ_genome 11227	Bacteroides thetaiotaomicron	MAG	GII	99.8	99	96.7	97.6	98.2	98.8
GUT_GENOME003489	Bacteroides thetaiotaomicron	MAG	GII	99.8	99	96.7	97.4	98.2	98.8
GUT_GENOME101535	Bacteroides thetaiotaomicron	MAG	GII	99.4	99	96.7	97.4	98.2	98.7
KIJ_genome 26710	Bacteroides uniformis	MAG	GII	99.8	99	96.7	97.4	98.2	98.8
KIJ_genome 6460	Bacteroides uniformis	MAG	GII	99.8	99	96.7	97.4	98.2	98.8
KIJ_genome 9193	Bacteroides uniformis	MAG	GII	99.4	99	96.7	97.4	98.2	98.7
KIJ_genome 4327	Bacteroides uniformis	MAG	GII	99.4	99	96.3	97.6	98.2	98.7
KIJ_genome 4881	Bacteroides uniformis	MAG	GII	99.4	99	96.3	97.6	98.2	98.7
GUT_GENOME001008	Bacteroides uniformis AF21-53	ISO	GII	99.8	99	96.7	97.4	98.2	98.8
GUT_GENOME001076	Bacteroides uniformis AF25-38AC	ISO	GII	99.4	99	96.7	97.4	98.2	98.7
GUT_GENOME001081	Bacteroides uniformis AF26-10BH	ISO	GII	99.8	99	96.7	97.4	98.2	98.8
GUT_GENOME001798	Bacteroides uniformis AM43-9	ISO	GII	99.8	99	96.7	97.6	98.2	98.8
GUT_GENOME016334	Bacteroides uniformis	MAG	GII	99.4	99	96.7	97.4	98.2	98.7
GUT_GENOME033675	Bacteroides uniformis	MAG	GII	99.4	99	96.3	97.6	98.2	98.6
GUT_GENOME147943	Bacteroides uniformis	MAG	GII	99.4	99	96.7	97.6	98.2	98.7
GUT_GENOME150364	Bacteroides uniformis	MAG	GII	99.4	99	96.7	97.4	98.2	98.7
GUT_GENOME151214	Bacteroides uniformis	MAG	GII	99.8	99	96.7	97.6	98.2	98.8
GUT_GENOME178496	Bacteroides uniformis	MAG	GII	99.4	99	96.7	97.4	98.2	98.7
GUT_GENOME227045	Bacteroides uniformis	MAG	GII	99.4	99	96.7	97.4	98.2	98.7
KIJ_genome 1091	Bacteroides vulgatus	MAG	GII	99.8	99	96.7	97.4	98.2	98.8
KIJ_genome 12389	Bacteroides vulgatus	MAG	GII	99.8	99	96.7	97.6	98.2	98.8
KIJ_genome 15885	Bacteroides vulgatus	MAG	GII	99.8	99	96.7	97.2	98.1	98.8
KIJ_genome 17209	Bacteroides vulgatus	MAG	GII	99.8	99	96.3	97.6	98.1	98.8
KIJ_genome 10033	Bacteroides vulgatus	MAG	GII	99.4	99	96.7	97.2	98.2	98.7
KIJ_genome 20177	Bacteroides vulgatus	MAG	GII	99.4	99	96.3	97.6	98.2	98.7
KIJ_genome 22411	Bacteroides xylanisolvens	MAG	GII	99.4	99	96.3	97.6	98.2	98.7
GUT_GENOME001249	Bacteroides xylanisolvens AF38-2	ISO	GII	99.4	99	96.7	97.2	98.2	98.7
GUT_GENOME153813	Bacteroides xylanisolvens	MAG	GII	99.8	99	96.7	97.4	98.2	98.8
KIJ_genome 22412	Bacteroides xylanisolvens	MAG	GII	99.8	99	96.7	97.4	98.1	98.8
KIJ_genome 15171	Parabacteroides johnsonii	MAG	GII	99.4	99	96.7	97.2	98.2	98.7
KIJ_genome 17018	Parabacteroides merdae	MAG	GII	99.8	99	96.7	97.4	98.2	98.8
GUT_GENOME099480	Tyzzerella nexilis	MAG	GII	99.4	99	96.7	97.2	98.2	98.7
GUT_GENOME085027	Bacteroides coprocola	MAG	GIIIrec (GI)	98.3	97.6	95.7	99.8	99.9	99.9
GUT_GENOME033354	Bacteroides dorei	MAG	GIIIrec (GI)	98.3	97.6	95.7	99.8	99.9	99.9
GUT_GENOME001331	Bacteroides plebeius AM09-36	ISO	GIIIrec (GI)	98.3	97.6	95.7	99.8	99.9	99.9
GUT_GENOME017313	Bacteroides coprocola	MAG	GIIIrec (GI)	98.3	98	93	99.8	99.9	99.9
GUT_GENOME150750	Bacteroides plebeius	MAG	GIIIrec (GI)	98.3	98	93	99.8	99.9	99.9
GUT_GENOME249569	Bacteroides plebeius	MAG	GIIIrec (GI)	98.3	98	93	98.7	99.1	99.9
KIJ_genome 9348	Bacteroides plebeius	MAG	GIIIrec (GII)	98.3	97.6	92	97.4	98.2	98.8
KIJ_genome 101	Bacteroides coprocola	MAG	GIII	98.3	97.5	92	96.3	97.9	96
KIJ_genome 26984	Bacteroides coprocola	MAG	GIII	98.3	97.7	92	96.3	97.9	96
KIJ_genome 15823	Bacteroides coprocola	MAG	GIII	98.3	97.7	92.3	96.1	97.9	95.9
GUT_GENOME032023	Bacteroides coprocola	MAG	GIII	98.3	97.7	92	96.3	97.9	96
GUT_GENOME044522	Bacteroides coprocola	MAG	GIII	98.3	97.6	92.3	96.3	97.9	96
KIJ_genome 7396	Bacteroides plebeius	MAG	GIII	98.5	98.1	92.3	97.1	97.9	96

KIJ_genome 15369	Bacteroides plebeius	MAG	GIII	98.3	97.6	92.3	96.3	98	96
KIJ_genome 9284	Bacteroides plebeius	MAG	GIII	98.3	97.6	92.3	96.1	97.9	96
KIJ_genome 24917	Bacteroides plebeius	MAG	GIII	98.3	97.7	92.3	96.3	97.8	96
KIJ_genome 19050	Bacteroides plebeius	MAG	GIII	98.3	97.6	92	96.3	97.9	96
KIJ_genome 22909	Bacteroides plebeius	MAG	GIII	98.3	97.6	92	96.1	97.9	96
KIJ_genome 26757	Bacteroides plebeius	MAG	GIII	98.3	97.6	92	96.3	97.9	96
GUT_GENOME000762	Bacteroides stercoris AF05-4	ISO	GIII	98.3	97.6	92.3	96.3	97.9	96
KIJ_genome 20422	Bacteroides vulgatus	MAG	GIII	98.3	97.6	92.3	96.3	97.9	96
KIJ_genome 24180	Bacteroides vulgatus	MAG	GIII	98.3	97.5	92	96.3	97.9	96
KIJ_genome 24692	Holdemanella sp002299315	MAG	GIII	98.3	97.7	92.3	97.2	97.9	96.2

Clade GI
Clade GII
Clade GIII
Outgroup
