# Supplementary material for: The porphyran degradation system is complete, phylogenetically and geographically diverse across the gut microbiota of East Asian populations
Source: PLoS One. 2025 Aug 1;20(8):e0329457. doi: 10.1371/journal.pone.0329457 (PMC12316285; doi:10.1371/journal.pone.0329457)
Supplement: S4 Table — (PDF) [file pone.0329457.s014.pdf]

Table S4: List of assembly projects probed with the genes encoding *PUL-PorB* and list of SRA projects probed with the 50 nucleotides probes.

Location of sampling		Project (assembly)	Bio-samples	PUL porB		Project (SRA)	Bio-samples	50 n probes	
				hits	%			hits	%
**CHINA**	Hangzhou	PRJEB24527	97	39	40	PRJNA375935	211	125	59
	Hangzhou	PRJEB29103	168	71	42	PRJNA356102	168	106	63
	Hangzhou	PRJEB26158	97	40	41	PRJNA353560	97	54	56
	Hangzhou					PRJEB6337	312	137	44
	Hangzhou					PRJNA505228	50	27	54
	Shenzen	PRJEB30046	370	135	36	PRJNA422434	370	163	44
	Shenzen	PRJEB26908	19	2	11	PRJEB12669	20	7	35
	Guangzhou					PRJEB18755	124	40	32
	Guangzhou					PRJEB15371	122	58	48
	Beijin					PRJNA401977	145	101	70
	Tangshan					PRJEB13870	193	62	32
	Hong-Kong	PRJEB24748	128	23	18	PRJNA557323	564	356	63
	Hunan					PRJNA349463	40	8	20
	Many places*	PRJEB26167	150	32	21	PRJNA356225	150	67	45
	Inner Mongolia					PRJNA328899	47	5	11
**MONGOLIA**	Ulan Bator/ Khentti / TUV province					PRJNA328899	63	2	3
**JAPAN**	Tokyo	PRJEB26092	254	50	20	PRJDB3601	255	133	52
	Kanazawa					PRJNA517801	68	10	15
	Tokyo					PRJDB7378	50	47	94
	Tokyo	PRJDB4176	643	170	26	PRJDB4176	645	548	85
**KOREA**	Seoul	PRJNA678426	90	23	26	PRJNA678426	106	96	91
	NA					PRJEB17896	27	24	89
**MALAYSIA**	Kuala Lumpur					PRJNA797994	56	9	16
	Orang Asli					PRJNA797994	351	3	1
**INDIA**	Bhopal	PRJEB33179	53	0	0	PRJNA397112	53	0	0
	Kasaragod	PRJEB33179	57	0	0	PRJNA397112	57	0	0
**BANGLADESH**	Dhaka	PRJEB22359	7	0	0				
**USA**	Lincoln	PRJEB26490	63	1	1.6	PRJNA324129	87	1	1
	Seattle	PRJEB22365	22	0	0				
	Saint Louis	PRJEB24849	387	0	0				
	HMP	PRJEB22283	717	5	0.7				
	Cheyenne tribe	PRJEB22552	37	0	0				
**PERU+USA**		PRJEB24847	36+11	0	0	PRJNA268964	36	0	0
**MADAGASCAR**		PREJB33766	112	0	0	PRJNA485056	112	0	0
**TANZANIA + ITALY**		PREJB22391	27+11	0	0	PRJNA278393	27+11	0	0
