# Supplementary material for: The porphyran degradation system is complete, phylogenetically and geographically diverse across the gut microbiota of East Asian populations
Source: PLoS One. 2025 Aug 1;20(8):e0329457. doi: 10.1371/journal.pone.0329457 (PMC12316285; doi:10.1371/journal.pone.0329457)

Table S5: List of the reconstructed *PUL-PorB* found in the assembled human gut metagenomes of Chineses, Japaneses and Korean populations. Only full length genes were reported in the list. The genes were classified in Group I , II, III or IIIrec based on their homology with those found in reference bacteria (Figure 4).

Study	Location	Biosample	GH29	GH16	SusC	SusD	GH2
PRJEB24527	Hangzhou (China)	SAMN06348158	GI	GI	GI	GI	GI
PRJEB24527	Hangzhou (China)	SAMN06348180		GI	GI	GI	GI
PRJEB24527	Hangzhou (China)	SAMN06348180	GI				
PRJEB24527	Hangzhou (China)	SAMN06348127	GIII	GIII	GI		
PRJEB24527	Hangzhou (China)	SAMN06348139	GII	GII	GII	GII	GII
PRJEB24527	Hangzhou (China)	SAMN06348137	GII	GII	GII	GII	GII
PRJEB24527	Hangzhou (China)	SAMN06348159		GII	GII		
PRJEB24527	Hangzhou (China)	SAMN06348159	GII				
PRJEB24527	Hangzhou (China)	SAMN06348194	GI	GI	GI		
PRJEB24527	Hangzhou (China)	SAMN06348194					GI
PRJEB24527	Hangzhou (China)	SAMN06348136	GIII	GIIIrec	GI	GI	GI
PRJEB24527	Hangzhou (China)	SAMN06348110					GII
PRJEB24527	Hangzhou (China)	SAMN06348110	GII	GII	GII	GII	
PRJEB24527	Hangzhou (China)	SAMN06348142		GI			
PRJEB24527	Hangzhou (China)	SAMN06348196	GI		GI	GI	GI
PRJEB24527	Hangzhou (China)	SAMN06348117	GI				
PRJEB24527	Hangzhou (China)	SAMN06348117		GI	GI	GI	GI
PRJEB24527	Hangzhou (China)	SAMN06348105	GIII	GIIIrec	GI	GI	GI
PRJEB24527	Hangzhou (China)	SAMN06348134	GI	GI	GI	GI	GI
PRJEB24527	Hangzhou (China)	SAMN06348145	GII	GII	GII		
PRJEB24527	Hangzhou (China)	SAMN06348167	GII	GII	GII	GII	
PRJEB24527	Hangzhou (China)	SAMN06348115	GI	GI	GI	GI	GI
PRJEB24527	Hangzhou (China)	SAMN06348140		GI			
PRJEB24527	Hangzhou (China)	SAMN06348168	GIII	GIII	GI	GI	GI
PRJEB24527	Hangzhou (China)	SAMN06348186	GII	GII	GII	GII	GII
PRJEB24527	Hangzhou (China)	SAMN06348183		GI			
PRJEB24527	Hangzhou (China)	SAMN06348193	GI		GI	GI	GI
PRJEB24527	Hangzhou (China)	SAMN06348190	GI	GI	GI	GI	GI
PRJEB24527	Hangzhou (China)	SAMN06348123	GII	GII	GII	GII	GII
PRJEB24527	Hangzhou (China)	SAMN06348149					GI
PRJEB24527	Hangzhou (China)	SAMN06348149	GI	GI	GI		
PRJEB24527	Hangzhou (China)	SAMN06348164	GI				
PRJEB24527	Hangzhou (China)	SAMN06348164					GI
PRJEB24527	Hangzhou (China)	SAMN06348164		GI	GI		
PRJEB24527	Hangzhou (China)	SAMN06348124	GI	GI			
PRJEB24527	Hangzhou (China)	SAMN06348124				GI	
PRJEB24527	Hangzhou (China)	SAMN06348192	GII	GII	GII		
PRJEB24527	Hangzhou (China)	SAMN06348176	GII				
PRJEB24527	Hangzhou (China)	SAMN06348176		GI	GI		
PRJEB24527	Hangzhou (China)	SAMN06348185	GI				
PRJEB24527	Hangzhou (China)	SAMN06348143					GI
PRJEB24527	Hangzhou (China)	SAMN06348156	GII	GII	GII	GII	GII
PRJEB24527	Hangzhou (China)	SAMN06348175	GII	GII	GII		
PRJEB24527	Hangzhou (China)	SAMN06348175					GII
PRJEB24527	Hangzhou (China)	SAMN06348152		GI	GI		
PRJEB24527	Hangzhou (China)	SAMN06348174			GI	GI	GI
PRJEB24527	Hangzhou (China)	SAMN06348150	GIII	GIIIrec	GI	GI	GI
PRJEB24527	Hangzhou (China)	SAMN06348113	GI	GI	GI		
PRJEB24527	Hangzhou (China)	SAMN06348166					GI
PRJEB24527	Hangzhou (China)	SAMN06348166	GI				
PRJEB24527	Hangzhou (China)	SAMN06348166		GI	GI		
PRJEB24527	Hangzhou (China)	SAMN06348146	GIII	GIII	GI	GI	GI
PRJEB26158	Hangzhou (China)	SAMN06016365		GI	GI		

Legend: GI (blue), GII (red), GIIIrec (green), GIII (yellow), Outgroup (grey)

Study	Location	Biosample	GH29	GH16	SusC	SusD	GH2
PRJEB26158	Hangzhou (China)	SAMN06016335					
PRJEB26158	Hangzhou (China)	SAMN06016335					
PRJEB26158	Hangzhou (China)	SAMN06016357					
PRJEB26158	Hangzhou (China)	SAMN06016417					
PRJEB26158	Hangzhou (China)	SAMN06016417					
PRJEB26158	Hangzhou (China)	SAMN06016417					
PRJEB26158	Hangzhou (China)	SAMN06016394					
PRJEB26158	Hangzhou (China)	SAMN06016376					
PRJEB26158	Hangzhou (China)	SAMN06016349					
PRJEB26158	Hangzhou (China)	SAMN06016375					
PRJEB26158	Hangzhou (China)	SAMN06016368					
PRJEB26158	Hangzhou (China)	SAMN06016400					
PRJEB26158	Hangzhou (China)	SAMN06016403					
PRJEB26158	Hangzhou (China)	SAMN06016395					
PRJEB26158	Hangzhou (China)	SAMN06016332					
PRJEB26158	Hangzhou (China)	SAMN06016333					
PRJEB26158	Hangzhou (China)	SAMN06016407					
PRJEB26158	Hangzhou (China)	SAMN06016369					
PRJEB26158	Hangzhou (China)	SAMN06016356					
PRJEB26158	Hangzhou (China)	SAMN06016341					
PRJEB26158	Hangzhou (China)	SAMN06016327					
PRJEB26158	Hangzhou (China)	SAMN06016354					
PRJEB26158	Hangzhou (China)	SAMN06016409					
PRJEB26158	Hangzhou (China)	SAMN06016409					
PRJEB26158	Hangzhou (China)	SAMN06016383					
PRJEB26158	Hangzhou (China)	SAMN06016383					
PRJEB26158	Hangzhou (China)	SAMN06016384					
PRJEB26158	Hangzhou (China)	SAMN06016384					
PRJEB26158	Hangzhou (China)	SAMN06016415					
PRJEB26158	Hangzhou (China)	SAMN06016420					
PRJEB26158	Hangzhou (China)	SAMN06016343					
PRJEB26158	Hangzhou (China)	SAMN06016411					
PRJEB26158	Hangzhou (China)	SAMN06016355					
PRJEB26158	Hangzhou (China)	SAMN06016355					
PRJEB26158	Hangzhou (China)	SAMN06016416					
PRJEB26158	Hangzhou (China)	SAMN06016416					
PRJEB26158	Hangzhou (China)	SAMN06016351					
PRJEB26158	Hangzhou (China)	SAMN06016377					
PRJEB26158	Hangzhou (China)	SAMN06016377					
PRJEB26158	Hangzhou (China)	SAMN06016406					
PRJEB26158	Hangzhou (China)	SAMN06016412					
PRJEB26158	Hangzhou (China)	SAMN06016412					
PRJEB26158	Hangzhou (China)	SAMN06016352					
PRJEB26158	Hangzhou (China)	SAMN06016389					
PRJEB26158	Hangzhou (China)	SAMN06016360					
PRJEB26158	Hangzhou (China)	SAMN06016391					
PRJEB26158	Hangzhou (China)	SAMN06016391					
PRJEB26158	Hangzhou (China)	SAMN06016362					
PRJEB26158	Hangzhou (China)	SAMN06016340					
PRJEB26158	Hangzhou (China)	SAMN06016364					
PRJEB29103	Hangzhou (China)	SAMN06111977					
PRJEB29103	Hangzhou (China)	SAMN06111977					
PRJEB29103	Hangzhou (China)	SAMN06112032					
PRJEB29103	Hangzhou (China)	SAMN06112032					
PRJEB29103	Hangzhou (China)	SAMN06112020					
PRJEB29103	Hangzhou (China)	SAMN06112008					
PRJEB29103	Hangzhou (China)	SAMN06112008					
PRJEB29103	Hangzhou (China)	SAMN06112024					

Study	Location	Biosample	GH29	GH16	SusC	SusD	GH2
PRJEB29103	Hangzhou (China)	SAMN06111979					
PRJEB29103	Hangzhou (China)	SAMN06112084					
PRJEB29103	Hangzhou (China)	SAMN06112084					
PRJEB29103	Hangzhou (China)	SAMN06112031					
PRJEB29103	Hangzhou (China)	SAMN06112031					
PRJEB29103	Hangzhou (China)	SAMN06112010					
PRJEB29103	Hangzhou (China)	SAMN06112010					
PRJEB29103	Hangzhou (China)	SAMN06112010					
PRJEB29103	Hangzhou (China)	SAMN06111975					
PRJEB29103	Hangzhou (China)	SAMN06111975					
PRJEB29103	Hangzhou (China)	SAMN06112021					
PRJEB29103	Hangzhou (China)	SAMN06112021					
PRJEB29103	Hangzhou (China)	SAMN06112021					
PRJEB29103	Hangzhou (China)	SAMN06111969					
PRJEB29103	Hangzhou (China)	SAMN06111969					
PRJEB29103	Hangzhou (China)	SAMN06111989					
PRJEB29103	Hangzhou (China)	SAMN06111989					
PRJEB29103	Hangzhou (China)	SAMN06112051					
PRJEB29103	Hangzhou (China)	SAMN06111957					
PRJEB29103	Hangzhou (China)	SAMN06705192					
PRJEB29103	Hangzhou (China)	SAMN06111983					
PRJEB29103	Hangzhou (China)	SAMN06111992					
PRJEB29103	Hangzhou (China)	SAMN06111992					
PRJEB29103	Hangzhou (China)	SAMN06111961					
PRJEB29103	Hangzhou (China)	SAMN06112053					
PRJEB29103	Hangzhou (China)	SAMN06111984					
PRJEB29103	Hangzhou (China)	SAMN06111995					
PRJEB29103	Hangzhou (China)	SAMN06111995					
PRJEB29103	Hangzhou (China)	SAMN06112015					
PRJEB29103	Hangzhou (China)	SAMN06111970					
PRJEB29103	Hangzhou (China)	SAMN06111970					
PRJEB29103	Hangzhou (China)	SAMN06111970					
PRJEB29103	Hangzhou (China)	SAMN06111999					
PRJEB29103	Hangzhou (China)	SAMN06111999					
PRJEB29103	Hangzhou (China)	SAMN06111999					
PRJEB29103	Hangzhou (China)	SAMN06111931					
PRJEB29103	Hangzhou (China)	SAMN06112046					
PRJEB29103	Hangzhou (China)	SAMN06112027					
PRJEB29103	Hangzhou (China)	SAMN06112034					
PRJEB29103	Hangzhou (China)	SAMN06112034					
PRJEB29103	Hangzhou (China)	SAMN06112034					
PRJEB29103	Hangzhou (China)	SAMN06112000					
PRJEB29103	Hangzhou (China)	SAMN06112044					
PRJEB29103	Hangzhou (China)	SAMN06112044					
PRJEB29103	Hangzhou (China)	SAMN06112044					
PRJEB29103	Hangzhou (China)	SAMN06112057					
PRJEB29103	Hangzhou (China)	SAMN06111937					
PRJEB29103	Hangzhou (China)	SAMN06111937					
PRJEB29103	Hangzhou (China)	SAMN06111948					
PRJEB29103	Hangzhou (China)	SAMN06111948					
PRJEB29103	Hangzhou (China)	SAMN06112019					
PRJEB29103	Hangzhou (China)	SAMN06112019					
PRJEB29103	Hangzhou (China)	SAMN06111946					
PRJEB29103	Hangzhou (China)	SAMN06111946					
PRJEB29103	Hangzhou (China)	SAMN06112061					
PRJEB29103	Hangzhou (China)	SAMN06112061					
PRJEB29103	Hangzhou (China)	SAMN06111988					
PRJEB29103	Hangzhou (China)	SAMN06111990					
PRJEB29103	Hangzhou (China)	SAMN06111991					

Study	Location	Biosample	GH29	GH16	SusC	SusD	GH2
PRJEB29103	Hangzhou (China)	SAMN06111996					
PRJEB29103	Hangzhou (China)	SAMN06112052					
PRJEB29103	Hangzhou (China)	SAMN06112042					
PRJEB29103	Hangzhou (China)	SAMN06111962					
PRJEB29103	Hangzhou (China)	SAMN06111962					
PRJEB29103	Hangzhou (China)	SAMN06111963					
PRJEB29103	Hangzhou (China)	SAMN06112005					
PRJEB29103	Hangzhou (China)	SAMN06112050					
PRJEB29103	Hangzhou (China)	SAMN06112050					
PRJEB29103	Hangzhou (China)	SAMN06112002					
PRJEB29103	Hangzhou (China)	SAMN06111997					
PRJEB29103	Hangzhou (China)	SAMN06111934					
PRJEB29103	Hangzhou (China)	SAMN06111950					
PRJEB29103	Hangzhou (China)	SAMN06111943					
PRJEB29103	Hangzhou (China)	SAMN06111943					
PRJEB29103	Hangzhou (China)	SAMN06112094					
PRJEB29103	Hangzhou (China)	SAMN06112058					
PRJEB29103	Hangzhou (China)	SAMN06112040					
PRJEB29103	Hangzhou (China)	SAMN06112040					
PRJEB29103	Hangzhou (China)	SAMN06112001					
PRJEB29103	Hangzhou (China)	SAMN06112001					
PRJEB29103	Hangzhou (China)	SAMN06112017					
PRJEB29103	Hangzhou (China)	SAMN06112069					
PRJEB29103	Hangzhou (China)	SAMN06111971					
PRJEB29103	Hangzhou (China)	SAMN06111953					
PRJEB29103	Hangzhou (China)	SAMN06111945					
PRJEB29103	Hangzhou (China)	SAMN06111929					
PRJEB29103	Hangzhou (China)	SAMN06112083					
PRJEB29103	Hangzhou (China)	SAMN06111980					
PRJEB29103	Hangzhou (China)	SAMN06112060					
PRJEB29103	Hangzhou (China)	SAMN06112060					
PRJEB29103	Hangzhou (China)	SAMN06111981					
PRJEB29103	Hangzhou (China)	SAMN06111967					
PRJEB29103	Hangzhou (China)	SAMN06112070					
PRJEB29103	Hangzhou (China)	SAMN06112070					
PRJEB29103	Hangzhou (China)	SAMN06111986					
PRJEB29103	Hangzhou (China)	SAMN06112073					
PRJEB29103	Hangzhou (China)	SAMN06112012					
PRJEB29103	Hangzhou (China)	SAMN06112012					
PRJEB29103	Hangzhou (China)	SAMN06112036					
PRJEB24748	Hong Kong (China)	SAMEA3541511					
PRJEB24748	Hong Kong (China)	SAMEA3541481					
PRJEB24748	Hong Kong (China)	SAMEA3541543					
PRJEB24748	Hong Kong (China)	SAMEA3541539					
PRJEB24748	Hong Kong (China)	SAMEA3541586					
PRJEB24748	Hong Kong (China)	SAMEA3541586					
PRJEB24748	Hong Kong (China)	SAMEA3541592					
PRJEB24748	Hong Kong (China)	SAMEA3541577					
PRJEB24748	Hong Kong (China)	SAMEA3541522					
PRJEB24748	Hong Kong (China)	SAMEA3541551					
PRJEB24748	Hong Kong (China)	SAMEA3541559					
PRJEB24748	Hong Kong (China)	SAMEA3541570					
PRJEB24748	Hong Kong (China)	SAMEA3541556					
PRJEB24748	Hong Kong (China)	SAMEA3541516					
PRJEB24748	Hong Kong (China)	SAMEA3541553					
PRJEB24748	Hong Kong (China)	SAMEA3541580					
PRJEB24748	Hong Kong (China)	SAMEA3541519					
PRJEB24748	Hong Kong (China)	SAMEA3541493					

Study	Location	Biosample	GH29	GH16	SusC	SusD	GH2
PRJEB24748	Hong Kong (China)	SAMEA3541531					
PRJEB24748	Hong Kong (China)	SAMEA3541554					
PRJEB24748	Hong Kong (China)	SAMEA3541585					
PRJEB24748	Hong Kong (China)	SAMEA3541585					
PRJEB24748	Hong Kong (China)	SAMEA3541472					
PRJEB24748	Hong Kong (China)	SAMEA3541557					
PRJEB24748	Hong Kong (China)	SAMEA3541557					
PRJEB24748	Hong Kong (China)	SAMEA3541524					
PRJEB24748	Hong Kong (China)	SAMEA3541524					
PRJEB26908	Shenzen (China)	SAMN02841183					
PRJEB26908	Shenzen (China)	SAMEA4031715					
PRJEB30046	Shenzen (China)	SAMN00791943					
PRJEB30046	Shenzen (China)	SAMN00791936					
PRJEB30046	Shenzen (China)	SAMN00791921					
PRJEB30046	Shenzen (China)	SAMN00791910					
PRJEB30046	Shenzen (China)	SAMN00791920					
PRJEB30046	Shenzen (China)	SAMN00791914					
PRJEB30046	Shenzen (China)	SAMN00791914					
PRJEB30046	Shenzen (China)	SAMN00791914					
PRJEB30046	Shenzen (China)	SAMN00791915					
PRJEB30046	Shenzen (China)	SAMN00791915					
PRJEB30046	Shenzen (China)	SAMN00791927					
PRJEB30046	Shenzen (China)	SAMN00791927					
PRJEB30046	Shenzen (China)	SAMN00791913					
PRJEB30046	Shenzen (China)	SAMN00791937					
PRJEB30046	Shenzen (China)	SAMN00791934					
PRJEB30046	Shenzen (China)	SAMN00791919					
PRJEB30046	Shenzen (China)	SAMN00791918					
PRJEB30046	Shenzen (China)	SAMN00791911					
PRJEB30046	Shenzen (China)	SAMN00791911					
PRJEB30046	Shenzen (China)	SAMN00791911					
PRJEB30046	Shenzen (China)	SAMN00791904					
PRJEB30046	Shenzen (China)	SAMN00791923					
PRJEB30046	Shenzen (China)	SAMN00791907					
PRJEB30046	Shenzen (China)	SAMN00791931					
PRJEB30046	Shenzen (China)	SAMN00791912					
PRJEB30046	Shenzen (China)	SAMN00715199					
PRJEB30046	Shenzen (China)	SAMN00791909					
PRJEB30046	Shenzen (China)	SAMN00715191					
PRJEB30046	Shenzen (China)	SAMN00715184					
PRJEB30046	Shenzen (China)	SAMN00715161					
PRJEB30046	Shenzen (China)	SAMN00715215					
PRJEB30046	Shenzen (China)	SAMN00715158					
PRJEB30046	Shenzen (China)	SAMN00715187					
PRJEB30046	Shenzen (China)	SAMN00715170					
PRJEB30046	Shenzen (China)	SAMN00715242					
PRJEB30046	Shenzen (China)	SAMN00715163					
PRJEB30046	Shenzen (China)	SAMN00715163					
PRJEB30046	Shenzen (China)	SAMN00715203					
PRJEB30046	Shenzen (China)	SAMN00715203					
PRJEB30046	Shenzen (China)	SAMN00715233					
PRJEB30046	Shenzen (China)	SAMN00715233					
PRJEB30046	Shenzen (China)	SAMN00715237					
PRJEB30046	Shenzen (China)	SAMN00715217					
PRJEB30046	Shenzen (China)	SAMN00715217					
PRJEB30046	Shenzen (China)	SAMN00715192					
PRJEB30046	Shenzen (China)	SAMN00715225					
PRJEB30046	Shenzen (China)	SAMN00715225					

Study	Location	Biosample	GH29	GH16	SusC	SusD	GH2
PRJEB30046	Shenzen (China)	SAMN00715196					
PRJEB30046	Shenzen (China)	SAMN00715231					
PRJEB30046	Shenzen (China)	SAMN00715168					
PRJEB30046	Shenzen (China)	SAMN00715168					
PRJEB30046	Shenzen (China)	SAMN00715239					
PRJEB30046	Shenzen (China)	SAMN00715238					
PRJEB30046	Shenzen (China)	SAMN00715174					
PRJEB30046	Shenzen (China)	SAMN00715197					
PRJEB30046	Shenzen (China)	SAMN00715224					
PRJEB30046	Shenzen (China)	SAMN00715171					
PRJEB30046	Shenzen (China)	SAMN00715241					
PRJEB30046	Shenzen (China)	SAMN00715176					
PRJEB30046	Shenzen (China)	SAMN00715173					
PRJEB30046	Shenzen (China)	SAMN00715220					
PRJEB30046	Shenzen (China)	SAMN00715234					
PRJEB30046	Shenzen (China)	SAMN00715219					
PRJEB30046	Shenzen (China)	SAMN00715156					
PRJEB30046	Shenzen (China)	SAMN00715156					
PRJEB30046	Shenzen (China)	SAMN00715148					
PRJEB30046	Shenzen (China)	SAMN00715137					
PRJEB30046	Shenzen (China)	SAMN00715136					
PRJEB30046	Shenzen (China)	SAMN00715145					
PRJEB30046	Shenzen (China)	SAMN00715139					
PRJEB30046	Shenzen (China)	SAMN00715143					
PRJEB30046	Shenzen (China)	SAMN00715143					
PRJEB30046	Shenzen (China)	SAMN00715134					
PRJEB30046	Shenzen (China)	SAMN00792025					
PRJEB30046	Shenzen (China)	SAMN00792038					
PRJEB30046	Shenzen (China)	SAMN00792008					
PRJEB30046	Shenzen (China)	SAMN00791959					
PRJEB30046	Shenzen (China)	SAMN00791969					
PRJEB30046	Shenzen (China)	SAMN00792036					
PRJEB30046	Shenzen (China)	SAMN00791998					
PRJEB30046	Shenzen (China)	SAMN00792026					
PRJEB30046	Shenzen (China)	SAMN00791946					
PRJEB30046	Shenzen (China)	SAMN00792016					
PRJEB30046	Shenzen (China)	SAMN00792011					
PRJEB30046	Shenzen (China)	SAMN00791961					
PRJEB30046	Shenzen (China)	SAMN00792029					
PRJEB30046	Shenzen (China)	SAMN00792043					
PRJEB30046	Shenzen (China)	SAMN00791953					
PRJEB30046	Shenzen (China)	SAMN00792017					
PRJEB30046	Shenzen (China)	SAMN00792035					
PRJEB30046	Shenzen (China)	SAMN00792007					
PRJEB30046	Shenzen (China)	SAMN00792042					
PRJEB30046	Shenzen (China)	SAMN00792031					
PRJEB30046	Shenzen (China)	SAMN00792031					
PRJEB30046	Shenzen (China)	SAMN00792022					
PRJEB30046	Shenzen (China)	SAMN00791990					
PRJEB30046	Shenzen (China)	SAMN00791984					
PRJEB30046	Shenzen (China)	SAMN00792028					
PRJEB30046	Shenzen (China)	SAMN00791967					
PRJEB30046	Shenzen (China)	SAMN00791968					
PRJEB30046	Shenzen (China)	SAMN00792020					
PRJEB30046	Shenzen (China)	SAMN00792039					
PRJEB30046	Shenzen (China)	SAMN00791952					
PRJEB30046	Shenzen (China)	SAMN00792027					
PRJEB30046	Shenzen (China)	SAMN00792027					

Study	Location	Biosample	GH29	GH16	SusC	SusD	GH2
PRJEB30046	Shenzen (China)	SAMN00792037					
PRJEB30046	Shenzen (China)	SAMN00715258					
PRJEB30046	Shenzen (China)	SAMN00715271					
PRJEB30046	Shenzen (China)	SAMN00715275					
PRJEB30046	Shenzen (China)	SAMN00715273					
PRJEB30046	Shenzen (China)	SAMN00715259					
PRJEB30046	Shenzen (China)	SAMN00715256					
PRJEB30046	Shenzen (China)	SAMN00715266					
PRJEB30046	Shenzen (China)	SAMN00715264					
PRJEB30046	Shenzen (China)	SAMN00715264					
PRJEB30046	Shenzen (China)	SAMN00715272					
PRJEB30046	Shenzen (China)	SAMN00715269					
PRJEB30046	Shenzen (China)	SAMN00715255					
PRJEB30046	Shenzen (China)	SAMN00715257					
PRJEB30046	Shenzen (China)	SAMN00792081					
PRJEB30046	Shenzen (China)	SAMN00792091					
PRJEB30046	Shenzen (China)	SAMN00792045					
PRJEB30046	Shenzen (China)	SAMN00792045					
PRJEB30046	Shenzen (China)	SAMN00792098					
PRJEB30046	Shenzen (China)	SAMN00792111					
PRJEB30046	Shenzen (China)	SAMN00792069					
PRJEB30046	Shenzen (China)	SAMN00792069					
PRJEB30046	Shenzen (China)	SAMN00792092					
PRJEB30046	Shenzen (China)	SAMN00792092					
PRJEB30046	Shenzen (China)	SAMN00792085					
PRJEB30046	Shenzen (China)	SAMN00792085					
PRJEB30046	Shenzen (China)	SAMN00792115					
PRJEB30046	Shenzen (China)	SAMN00792062					
PRJEB30046	Shenzen (China)	SAMN00792107					
PRJEB30046	Shenzen (China)	SAMN00792073					
PRJEB30046	Shenzen (China)	SAMN00792112					
PRJEB30046	Shenzen (China)	SAMN00792061					
PRJEB30046	Shenzen (China)	SAMN00792048					
PRJEB30046	Shenzen (China)	SAMN00792099					
PRJEB30046	Shenzen (China)	SAMN00792104					
PRJEB30046	Shenzen (China)	SAMN00792058					
PRJEB30046	Shenzen (China)	SAMN00792080					
PRJEB30046	Shenzen (China)	SAMN00792080					
PRJEB30046	Shenzen (China)	SAMN00792084					
PRJEB30046	Shenzen (China)	SAMN00792089					
PRJEB30046	Shenzen (China)	SAMN00792089					
PRJEB30046	Shenzen (China)	SAMN00792093					
PRJEB30046	Shenzen (China)	SAMN00792056					
PRJEB30046	Shenzen (China)	SAMN00792079					
PRJEB30046	Shenzen (China)	SAMN00792047					
PRJEB30046	Shenzen (China)	SAMN00792102					
PRJEB30046	Shenzen (China)	SAMN00792070					
PRJEB30046	Shenzen (China)	SAMN00792053					
PRJEB30046	Shenzen (China)	SAMN00792057					
PRJEB30046	Shenzen (China)	SAMN00792055					
PRJEB30046	Shenzen (China)	SAMN00792067					
PRJEB30046	Shenzen (China)	SAMN00993245					
PRJEB30046	Shenzen (China)	SAMN00993245					
PRJEB30046	Shenzen (China)	SAMN00993243					
PRJEB26092	Tokyo (Japan)	SAMD00036237					
PRJEB26092	Tokyo (Japan)	SAMD00036296					
PRJEB26092	Tokyo (Japan)	SAMD00036296					
PRJEB26092	Tokyo (Japan)	SAMD00036241					

Study	Location	Biosample	GH29	GH16	SusC	SusD	GH2
PRJEB26092	Tokyo (Japan)	SAMD00036241					
PRJEB26092	Tokyo (Japan)	SAMD00036237					
PRJEB26092	Tokyo (Japan)	SAMD00036298					
PRJEB26092	Tokyo (Japan)	SAMD00036284					
PRJEB26092	Tokyo (Japan)	SAMD00036244					
PRJEB26092	Tokyo (Japan)	SAMD00036252					
PRJEB26092	Tokyo (Japan)	SAMD00036252					
PRJEB26092	Tokyo (Japan)	SAMD00036252					
PRJEB26092	Tokyo (Japan)	SAMD00036244					
PRJEB26092	Tokyo (Japan)	SAMD00036244					
PRJEB26092	Tokyo (Japan)	SAMD00036252					
PRJEB26092	Tokyo (Japan)	SAMD00036242					
PRJEB26092	Tokyo (Japan)	SAMD00036242					
PRJEB26092	Tokyo (Japan)	SAMD00036238					
PRJEB26092	Tokyo (Japan)	SAMD00036230					
PRJEB26092	Tokyo (Japan)	SAMD00036296					
PRJEB26092	Tokyo (Japan)	SAMD00036296					
PRJEB26092	Tokyo (Japan)	SAMD00036297					
PRJEB26092	Tokyo (Japan)	SAMD00036297					
PRJEB26092	Tokyo (Japan)	SAMD00036305					
PRJEB26092	Tokyo (Japan)	SAMD00036320					
PRJEB26092	Tokyo (Japan)	SAMD00036344					
PRJEB26092	Tokyo (Japan)	SAMD00036337					
PRJEB26092	Tokyo (Japan)	SAMD00036308					
PRJEB26092	Tokyo (Japan)	SAMD00036308					
PRJEB26092	Tokyo (Japan)	SAMD00036328					
PRJEB26092	Tokyo (Japan)	SAMD00036320					
PRJEB26092	Tokyo (Japan)	SAMD00036204					
PRJEB26092	Tokyo (Japan)	SAMD00036202					
PRJEB26092	Tokyo (Japan)	SAMD00036197					
PRJEB26092	Tokyo (Japan)	SAMD00036197					
PRJEB26092	Tokyo (Japan)	SAMD00036213					
PRJEB26092	Tokyo (Japan)	SAMD00036204					
PRJEB26092	Tokyo (Japan)	SAMD00036192					
PRJEB26092	Tokyo (Japan)	SAMD00036210					
PRJEB26092	Tokyo (Japan)	SAMD00036195					
PRJEB26092	Tokyo (Japan)	SAMD00036195					
PRJEB26092	Tokyo (Japan)	SAMD00036215					
PRJEB26092	Tokyo (Japan)	SAMD00036197					
PRJEB26092	Tokyo (Japan)	SAMD00036215					
PRJEB26092	Tokyo (Japan)	SAMD00036197					
PRJEB26092	Tokyo (Japan)	SAMD00036197					
PRJEB26092	Tokyo (Japan)	SAMD00036441					
PRJEB26092	Tokyo (Japan)	SAMD00036203					
PRJEB26092	Tokyo (Japan)	SAMD00036203					
PRJEB26092	Tokyo (Japan)	SAMD00036214					
PRJEB26092	Tokyo (Japan)	SAMD00036217					
PRJEB26092	Tokyo (Japan)	SAMD00036217					
PRJEB26092	Tokyo (Japan)	SAMD00036215					
PRJEB26092	Tokyo (Japan)	SAMD00036192					
PRJEB26092	Tokyo (Japan)	SAMD00036210					
PRJEB26092	Tokyo (Japan)	SAMD00036215					
PRJEB26092	Tokyo (Japan)	SAMD00036192					
PRJEB26092	Tokyo (Japan)	SAMD00036441					
PRJEB26092	Tokyo (Japan)	SAMD00036441					
PRJEB26092	Tokyo (Japan)	SAMD00036436					
PRJEB26092	Tokyo (Japan)	SAMD00036436					
PRJEB26092	Tokyo (Japan)	SAMD00036214					

Study	Location	Biosample	GH29	GH16	SusC	SusD	GH2
PRJEB26092	Tokyo (Japan)	SAMD00036352					
PRJEB26092	Tokyo (Japan)	SAMD00036359					
PRJEB26092	Tokyo (Japan)	SAMD00036354					
PRJEB26092	Tokyo (Japan)	SAMD00036354					
PRJEB26092	Tokyo (Japan)	SAMD00036367					
PRJEB26092	Tokyo (Japan)	SAMD00036359					
PRJEB26092	Tokyo (Japan)	SAMD00036350					
PRJEB26092	Tokyo (Japan)	SAMD00036354					
PRJEB26092	Tokyo (Japan)	SAMD00036349					
PRJEB26092	Tokyo (Japan)	SAMD00036354					
PRJEB26092	Tokyo (Japan)	SAMD00036354					
PRJEB26092	Tokyo (Japan)	SAMD00036409					
PRJEB26092	Tokyo (Japan)	SAMD00036409					
PRJEB26092	Tokyo (Japan)	SAMD00036419					
PRJEB26092	Tokyo (Japan)	SAMD00036419					
PRJEB26092	Tokyo (Japan)	SAMD00036349					
PRJEB26092	Tokyo (Japan)	SAMD00036349					
PRJEB26092	Tokyo (Japan)	SAMD00036388					
PRJEB26092	Tokyo (Japan)	SAMD00036412					
PRJEB26092	Tokyo (Japan)	SAMD00036404					
PRJEB26092	Tokyo (Japan)	SAMD00036404					
PRJEB26092	Tokyo (Japan)	SAMD00036404					
PRJEB26092	Tokyo (Japan)	SAMD00036404					
PRJEB26092	Tokyo (Japan)	SAMD00036400					
PRJEB26092	Tokyo (Japan)	SAMD00036392					
PRJEB26092	Tokyo (Japan)	SAMD00036388					
PRJEB26092	Tokyo (Japan)	SAMD00036388					
PRJEB26092	Tokyo (Japan)	SAMD00036388					
PRJEB26092	Tokyo (Japan)	SAMD00036413					
PRJEB26092	Tokyo (Japan)	SAMD00036405					
PRJEB26092	Tokyo (Japan)	SAMD00036405					
PRJEB26092	Tokyo (Japan)	SAMD00036379					
PRJEB26092	Tokyo (Japan)	SAMD00036398					
PRJEB26092	Tokyo (Japan)	SAMD00036432					
PRJEB26092	Tokyo (Japan)	SAMD00036377					
PRJEB26092	Tokyo (Japan)	SAMD00036388					
PRJEB26092	Tokyo (Japan)	SAMD00036386					
PRJEB26092	Tokyo (Japan)	SAMD00036400					
PRJEB26092	Tokyo (Japan)	SAMD00036386					
PRJEB26092	Tokyo (Japan)	SAMD00036379					
PRJEB26092	Tokyo (Japan)	SAMD00036377					
PRJEB26092	Tokyo (Japan)	SAMD00036404					
PRJEB26092	Tokyo (Japan)	SAMD00036432					
PRJDB4176	Tokyo (Japan)	SAMD00114727					
PRJDB4176	Tokyo (Japan)	SAMD00114731					
PRJDB4176	Tokyo (Japan)	SAMD00114734					
PRJDB4176	Tokyo (Japan)	SAMD00114737					
PRJDB4176	Tokyo (Japan)	SAMD00114760					
PRJDB4176	Tokyo (Japan)	SAMD00114798					
PRJDB4176	Tokyo (Japan)	SAMD00114805					
PRJDB4176	Tokyo (Japan)	SAMD00114805					
PRJDB4176	Tokyo (Japan)	SAMD00114805					
PRJDB4176	Tokyo (Japan)	SAMD00114825					
PRJDB4176	Tokyo (Japan)	SAMD00114825					
PRJDB4176	Tokyo (Japan)	SAMD00114829					
PRJDB4176	Tokyo (Japan)	SAMD00114834					
PRJDB4176	Tokyo (Japan)	SAMD00114834					
PRJDB4176	Tokyo (Japan)	SAMD00114865					

Study	Location	Biosample	GH29	GH16	SusC	SusD	GH2
PRJDB4176	Tokyo (Japan)	SAMD00114871					
PRJDB4176	Tokyo (Japan)	SAMD00114892					
PRJDB4176	Tokyo (Japan)	SAMD00114893					
PRJDB4176	Tokyo (Japan)	SAMD00114895					
PRJDB4176	Tokyo (Japan)	SAMD00114953					
PRJDB4176	Tokyo (Japan)	SAMD00114954					
PRJDB4176	Tokyo (Japan)	SAMD00114966					
PRJDB4176	Tokyo (Japan)	SAMD00114977					
PRJDB4176	Tokyo (Japan)	SAMD00115001					
PRJDB4176	Tokyo (Japan)	SAMD00115023					
PRJDB4176	Tokyo (Japan)	SAMD00115023					
PRJDB4176	Tokyo (Japan)	SAMD00115023					
PRJDB4176	Tokyo (Japan)	SAMD00154995					
PRJDB4176	Tokyo (Japan)	SAMD00164695					
PRJDB4176	Tokyo (Japan)	SAMD00164713					
PRJDB4176	Tokyo (Japan)	SAMD00164721					
PRJDB4176	Tokyo (Japan)	SAMD00164755					
PRJDB4176	Tokyo (Japan)	SAMD00164756					
PRJDB4176	Tokyo (Japan)	SAMD00164765					
PRJDB4176	Tokyo (Japan)	SAMD00164767					
PRJDB4176	Tokyo (Japan)	SAMD00164769					
PRJDB4176	Tokyo (Japan)	SAMD00164778					
PRJDB4176	Tokyo (Japan)	SAMD00164780					
PRJDB4176	Tokyo (Japan)	SAMD00164781					
PRJDB4176	Tokyo (Japan)	SAMD00164805					
PRJDB4176	Tokyo (Japan)	SAMD00164808					
PRJDB4176	Tokyo (Japan)	SAMD00164817					
PRJDB4176	Tokyo (Japan)	SAMD00164817					
PRJDB4176	Tokyo (Japan)	SAMD00164817					
PRJDB4176	Tokyo (Japan)	SAMD00164818					
PRJDB4176	Tokyo (Japan)	SAMD00164819					
PRJDB4176	Tokyo (Japan)	SAMD00164820					
PRJDB4176	Tokyo (Japan)	SAMD00164824					
PRJDB4176	Tokyo (Japan)	SAMD00164829					
PRJDB4176	Tokyo (Japan)	SAMD00164830					
PRJDB4176	Tokyo (Japan)	SAMD00164832					
PRJDB4176	Tokyo (Japan)	SAMD00164833					
PRJDB4176	Tokyo (Japan)	SAMD00164833					
PRJDB4176	Tokyo (Japan)	SAMD00164834					
PRJDB4176	Tokyo (Japan)	SAMD00164835					
PRJDB4176	Tokyo (Japan)	SAMD00164841					
PRJDB4176	Tokyo (Japan)	SAMD00164841					
PRJDB4176	Tokyo (Japan)	SAMD00164841					
PRJDB4176	Tokyo (Japan)	SAMD00164849					
PRJDB4176	Tokyo (Japan)	SAMD00164853					
PRJDB4176	Tokyo (Japan)	SAMD00164856					
PRJDB4176	Tokyo (Japan)	SAMD00164860					
PRJDB4176	Tokyo (Japan)	SAMD00164860					
PRJDB4176	Tokyo (Japan)	SAMD00164863					
PRJDB4176	Tokyo (Japan)	SAMD00164867					
PRJDB4176	Tokyo (Japan)	SAMD00164867					
PRJDB4176	Tokyo (Japan)	SAMD00164869					
PRJDB4176	Tokyo (Japan)	SAMD00164872					
PRJDB4176	Tokyo (Japan)	SAMD00164874					
PRJDB4176	Tokyo (Japan)	SAMD00164888					
PRJDB4176	Tokyo (Japan)	SAMD00164889					
PRJDB4176	Tokyo (Japan)	SAMD00164894					
PRJDB4176	Tokyo (Japan)	SAMD00164895					

Study	Location	Biosample	GH29	GH16	SusC	SusD	GH2
PRJDB4176	Tokyo (Japan)	SAMD00164895					
PRJDB4176	Tokyo (Japan)	SAMD00164897					
PRJDB4176	Tokyo (Japan)	SAMD00164898					
PRJDB4176	Tokyo (Japan)	SAMD00164900					
PRJDB4176	Tokyo (Japan)	SAMD00164901					
PRJDB4176	Tokyo (Japan)	SAMD00164915					
PRJDB4176	Tokyo (Japan)	SAMD00164916					
PRJDB4176	Tokyo (Japan)	SAMD00164921					
PRJDB4176	Tokyo (Japan)	SAMD00164922					
PRJDB4176	Tokyo (Japan)	SAMD00164924					
PRJDB4176	Tokyo (Japan)	SAMD00164925					
PRJDB4176	Tokyo (Japan)	SAMD00164928					
PRJDB4176	Tokyo (Japan)	SAMD00164942					
PRJDB4176	Tokyo (Japan)	SAMD00164945					
PRJDB4176	Tokyo (Japan)	SAMD00164946					
PRJDB4176	Tokyo (Japan)	SAMD00164948					
PRJDB4176	Tokyo (Japan)	SAMD00164950					
PRJDB4176	Tokyo (Japan)	SAMD00164954					
PRJDB4176	Tokyo (Japan)	SAMD00164967					
PRJDB4176	Tokyo (Japan)	SAMD00164967					
PRJDB4176	Tokyo (Japan)	SAMD00164976					
PRJDB4176	Tokyo (Japan)	SAMD00164977					
PRJDB4176	Tokyo (Japan)	SAMD00164984					
PRJDB4176	Tokyo (Japan)	SAMD00164987					
PRJDB4176	Tokyo (Japan)	SAMD00164991					
PRJDB4176	Tokyo (Japan)	SAMD00164995					
PRJDB4176	Tokyo (Japan)	SAMD00164995					
PRJDB4176	Tokyo (Japan)	SAMD00164998					
PRJDB4176	Tokyo (Japan)	SAMD00164999					
PRJDB4176	Tokyo (Japan)	SAMD00165000					
PRJDB4176	Tokyo (Japan)	SAMD00165002					
PRJDB4176	Tokyo (Japan)	SAMD00165004					
PRJDB4176	Tokyo (Japan)	SAMD00165005					
PRJDB4176	Tokyo (Japan)	SAMD00165015					
PRJDB4176	Tokyo (Japan)	SAMD00165032					
PRJDB4176	Tokyo (Japan)	SAMD00114718					
PRJDB4176	Tokyo (Japan)	SAMD00114719					
PRJDB4176	Tokyo (Japan)	SAMD00114719					
PRJDB4176	Tokyo (Japan)	SAMD00114721					
PRJDB4176	Tokyo (Japan)	SAMD00114729					
PRJDB4176	Tokyo (Japan)	SAMD00114738					
PRJDB4176	Tokyo (Japan)	SAMD00114740					
PRJDB4176	Tokyo (Japan)	SAMD00114742					
PRJDB4176	Tokyo (Japan)	SAMD00114756					
PRJDB4176	Tokyo (Japan)	SAMD00114758					
PRJDB4176	Tokyo (Japan)	SAMD00114768					
PRJDB4176	Tokyo (Japan)	SAMD00114771					
PRJDB4176	Tokyo (Japan)	SAMD00114779					
PRJDB4176	Tokyo (Japan)	SAMD00114788					
PRJDB4176	Tokyo (Japan)	SAMD00114799					
PRJDB4176	Tokyo (Japan)	SAMD00114799					
PRJDB4176	Tokyo (Japan)	SAMD00114800					
PRJDB4176	Tokyo (Japan)	SAMD00114802					
PRJDB4176	Tokyo (Japan)	SAMD00114804					
PRJDB4176	Tokyo (Japan)	SAMD00114806					
PRJDB4176	Tokyo (Japan)	SAMD00114806					
PRJDB4176	Tokyo (Japan)	SAMD00114807					
PRJDB4176	Tokyo (Japan)	SAMD00114810					

Study	Location	Biosample	GH29	GH16	SusC	SusD	GH2
PRJDB4176	Tokyo (Japan)	SAMD00114816					
PRJDB4176	Tokyo (Japan)	SAMD00114819					
PRJDB4176	Tokyo (Japan)	SAMD00114824					
PRJDB4176	Tokyo (Japan)	SAMD00114828					
PRJDB4176	Tokyo (Japan)	SAMD00114833					
PRJDB4176	Tokyo (Japan)	SAMD00114847					
PRJDB4176	Tokyo (Japan)	SAMD00114851					
PRJDB4176	Tokyo (Japan)	SAMD00114853					
PRJDB4176	Tokyo (Japan)	SAMD00114853					
PRJDB4176	Tokyo (Japan)	SAMD00114856					
PRJDB4176	Tokyo (Japan)	SAMD00114870					
PRJDB4176	Tokyo (Japan)	SAMD00114872					
PRJDB4176	Tokyo (Japan)	SAMD00114872					
PRJDB4176	Tokyo (Japan)	SAMD00114877					
PRJDB4176	Tokyo (Japan)	SAMD00114878					
PRJDB4176	Tokyo (Japan)	SAMD00114885					
PRJDB4176	Tokyo (Japan)	SAMD00114885					
PRJDB4176	Tokyo (Japan)	SAMD00114888					
PRJDB4176	Tokyo (Japan)	SAMD00114891					
PRJDB4176	Tokyo (Japan)	SAMD00114896					
PRJDB4176	Tokyo (Japan)	SAMD00114897					
PRJDB4176	Tokyo (Japan)	SAMD00114903					
PRJDB4176	Tokyo (Japan)	SAMD00114904					
PRJDB4176	Tokyo (Japan)	SAMD00114909					
PRJDB4176	Tokyo (Japan)	SAMD00114913					
PRJDB4176	Tokyo (Japan)	SAMD00114919					
PRJDB4176	Tokyo (Japan)	SAMD00114922					
PRJDB4176	Tokyo (Japan)	SAMD00114927					
PRJDB4176	Tokyo (Japan)	SAMD00114927					
PRJDB4176	Tokyo (Japan)	SAMD00114928					
PRJDB4176	Tokyo (Japan)	SAMD00114928					
PRJDB4176	Tokyo (Japan)	SAMD00114939					
PRJDB4176	Tokyo (Japan)	SAMD00114940					
PRJDB4176	Tokyo (Japan)	SAMD00114947					
PRJDB4176	Tokyo (Japan)	SAMD00114961					
PRJDB4176	Tokyo (Japan)	SAMD00114962					
PRJDB4176	Tokyo (Japan)	SAMD00114968					
PRJDB4176	Tokyo (Japan)	SAMD00114981					
PRJDB4176	Tokyo (Japan)	SAMD00114984					
PRJDB4176	Tokyo (Japan)	SAMD00114987					
PRJDB4176	Tokyo (Japan)	SAMD00114989					
PRJDB4176	Tokyo (Japan)	SAMD00114990					
PRJDB4176	Tokyo (Japan)	SAMD00114993					
PRJDB4176	Tokyo (Japan)	SAMD00114999					
PRJDB4176	Tokyo (Japan)	SAMD00114999					
PRJDB4176	Tokyo (Japan)	SAMD00115004					
PRJDB4176	Tokyo (Japan)	SAMD00115005					
PRJDB4176	Tokyo (Japan)	SAMD00115005					
PRJDB4176	Tokyo (Japan)	SAMD00115006					
PRJDB4176	Tokyo (Japan)	SAMD00115006					
PRJDB4176	Tokyo (Japan)	SAMD00115007					
PRJDB4176	Tokyo (Japan)	SAMD00115010					
PRJDB4176	Tokyo (Japan)	SAMD00115015					
PRJDB4176	Tokyo (Japan)	SAMD00115015					
PRJDB4176	Tokyo (Japan)	SAMD00115018					
PRJDB4176	Tokyo (Japan)	SAMD00115024					
PRJDB4176	Tokyo (Japan)	SAMD00115027					
PRJDB4176	Tokyo (Japan)	SAMD00115036					

Study	Location	Biosample	GH29	GH16	SusC	SusD	GH2
PRJDB4176	Tokyo (Japan)	SAMD00115045					
PRJDB4176	Tokyo (Japan)	SAMD00115048					
PRJDB4176	Tokyo (Japan)	SAMD00115051					
PRJDB4176	Tokyo (Japan)	SAMD00115053					
PRJDB4176	Tokyo (Japan)	SAMD00115059					
PRJDB4176	Tokyo (Japan)	SAMD00115063					
PRJDB4176	Tokyo (Japan)	SAMD00115068					
PRJDB4176	Tokyo (Japan)	SAMD00115074					
PRJDB4176	Tokyo (Japan)	SAMD00115089					
PRJNA678426	Korea	SAMN16796161					
PRJNA678426	Korea	SAMN16796164					
PRJNA678426	Korea	SAMN16796151					
PRJNA678426	Korea	SAMN16796151					
PRJNA678426	Korea	SAMN16796157					
PRJNA678426	Korea	SAMN16796157					
PRJNA678426	Korea	SAMN16796165					
PRJNA678426	Korea	SAMN16796170					
PRJNA678426	Korea	SAMN16796172					
PRJNA678426	Korea	SAMN16796174					
PRJNA678426	Korea	SAMN16796176					
PRJNA678426	Korea	SAMN16796179					
PRJNA678426	Korea	SAMN16796183					
PRJNA678426	Korea	SAMN16796184					
PRJNA678426	Korea	SAMN16796188					
PRJNA678426	Korea	SAMN16796194					
PRJNA678426	Korea	SAMN16796195					
PRJNA678426	Korea	SAMN16796203					
PRJNA678426	Korea	SAMN16796204					
PRJNA678426	Korea	SAMN16796211					
PRJNA678426	Korea	SAMN16796213					
PRJNA678426	Korea	SAMN16796215					
PRJNA678426	Korea	SAMN16796217					
PRJNA678426	Korea	SAMN16796221					
PRJNA678426	Korea	SAMN16796236					
PRJEB26167	Anhui (China)	SAMN06115447					
PRJEB26167	Anhui (China)	SAMN06115484					
PRJEB26167	Anhui (China)	SAMN06115505					
PRJEB26167	Chongqing (China)	SAMN06115457					
PRJEB26167	Chongqing (China)	SAMN06115459					
PRJEB26167	Chongqing (China)	SAMN06115468					
PRJEB26167	Chongqing (China)	SAMN06115485					
PRJEB26167	Chongqing (China)	SAMN06115492					
PRJEB26167	Fujian (China)	SAMN06115453					
PRJEB26167	Gansu (China)	SAMN06115528					
PRJEB26167	Guizhou (China)	SAMN06115411					
PRJEB26167	Guizhou (China)	SAMN06115416					
PRJEB26167	Guizhou (China)	SAMN06115421					
PRJEB26167	Guizhou (China)	SAMN06115482					
PRJEB26167	Henan (China)	SAMN06115427					
PRJEB26167	Henan (China)	SAMN06115433					
PRJEB26167	Henan (China)	SAMN06115441					
PRJEB26167	Henan (China)	SAMN06115443					
PRJEB26167	Henan (China)	SAMN06115455					
PRJEB26167	Henan (China)	SAMN06115470					
PRJEB26167	Hubei (China)	SAMN06115440					
PRJEB26167	Hubei (China)	SAMN06115444					
PRJEB26167	Hubei (China)	SAMN06115501					
PRJEB26167	Jiangsu (China)	SAMN06115452					

PRJEB26167	Jiangsu (China)	SAMN06115474
PRJEB26167	Ningxia (China)	SAMN06115515
PRJEB26167	Shanxi (China)	SAMN06115514
PRJEB26167	Shanxi (China)	SAMN06115539
PRJEB26167	Sichuan (China)	SAMN06115478
PRJEB26167	Sichuan (China)	SAMN06115483
PRJEB26167	Sichuan (China)	SAMN06115510
PRJEB26167	Sichuan (China)	SAMN06115530
PRJEB22283	HMP (USA)	SAMN00034905
PRJEB22283	HMP (USA)	SAMN00037012
PRJEB22283	HMP (USA)	SAMN00038172
PRJEB22283	HMP (USA)	SAMN00045549
PRJEB22283	HMP (USA)	SAMN00045843
PRJEB26490	Lincoln (USA)	SAMN05194702
PRJEB26155	Chicago (USA)	SAMN06018971
PRJEB26155	Chicago (USA)	SAMN06018972
PRJEB26155	Chicago (USA)	SAMN06018973
PRJNA288562	Standford (USA)	SAMN09866713

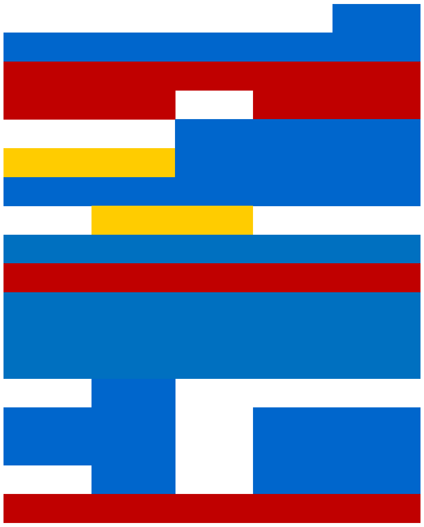

Supplement: S5 Table — Only full length genes were reported in the list. The genes were classified in Group I, II, III or IIIrec based on their homology with those found in reference bacteria (Fig 4). (PDF) [file pone.0329457.s015.pdf]
